# Supplementary material for: N-Formimidoylation/-iminoacetylation modification in aminoglycosides requires FAD-dependent and ligand-protein NOS bridge dual chemistry
Source: Nat Commun. 2023 May 3;14:2528. doi: 10.1038/s41467-023-38218-w (PMC10156733; doi:10.1038/s41467-023-38218-w)
Supplement: Supplementary file 1 — Supplementary Information [file 41467_2023_38218_MOESM1_ESM.pdf]

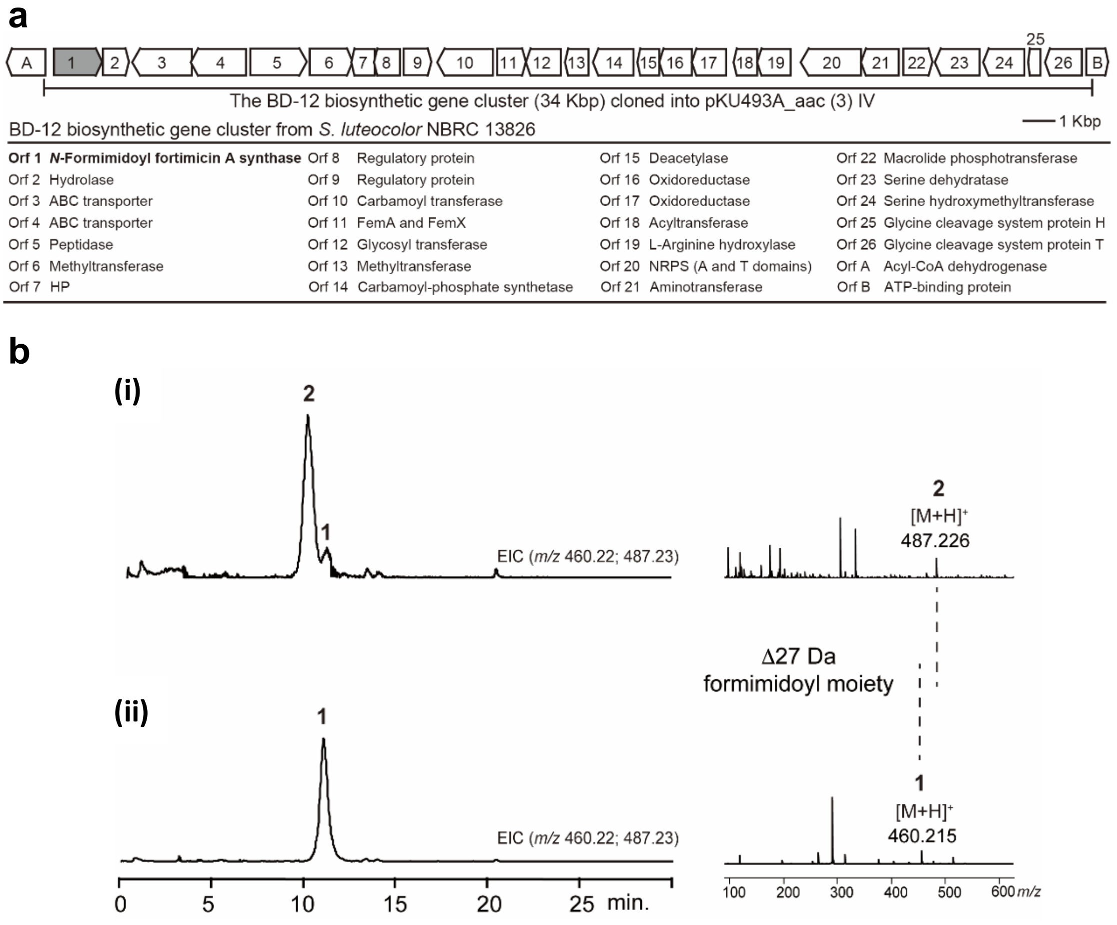

Supplementary Figure 1. Functional analysis of the *orf1* gene encoded in the BD-12 BGC from *S. luteocolor* NBRC 13826 (a BD-12 producer). (a) Gene organization of the BD-12-biosynthetic gene cluster (accession no. LC122485) from *S. luteocolor* NBRC 13826 is shown. The dark-gray-shaded gene (*orf1*) is the gene responsible for the *N*-formimidoyl group biosynthesis in BD-12. (b) The *S. avermitilis* SUKA17 transformant harboring pKU493A-BD-12 (wild gene cluster) (i) or pKU493A-BD-12-Δorf1 (ii) was cultivated. The culture broths were analyzed by HPLC-HR-ESI-MS using a phosphorylcholine-modified hydrophilic-interaction liquid chromatography (HILIC) column (SeQuant ZIC-cHILIC; 150 × 2.1 mm; Merck Millipore, Billerica, MA, USA) at 30°C at a flow rate of 0.3 ml min^{-1} and with a gradient of acetonitrile-water run over 30 min (70% (v/v) acetonitrile for 3 min, 70-40% (v/v) acetonitrile for 17 min, and 10% (v/v) for 10 min). The water contained 20 mM $HCOONH_4$ (pH 3.5). Extracted ion chromatograms (EICs) (left) for BD-12 (Fig. 1a, compound **2**) (m/z 487.23) and glycinothricin (Fig. 1a, compound **1**) (m/z 460.22) in the medium broths, and their MS spectra (right) are shown. The purified **1** was subjected to MS, NMR analysis (Supplementary Fig. 2) and assayed with Orf1 (Supplementary Fig. 31a, b).

a

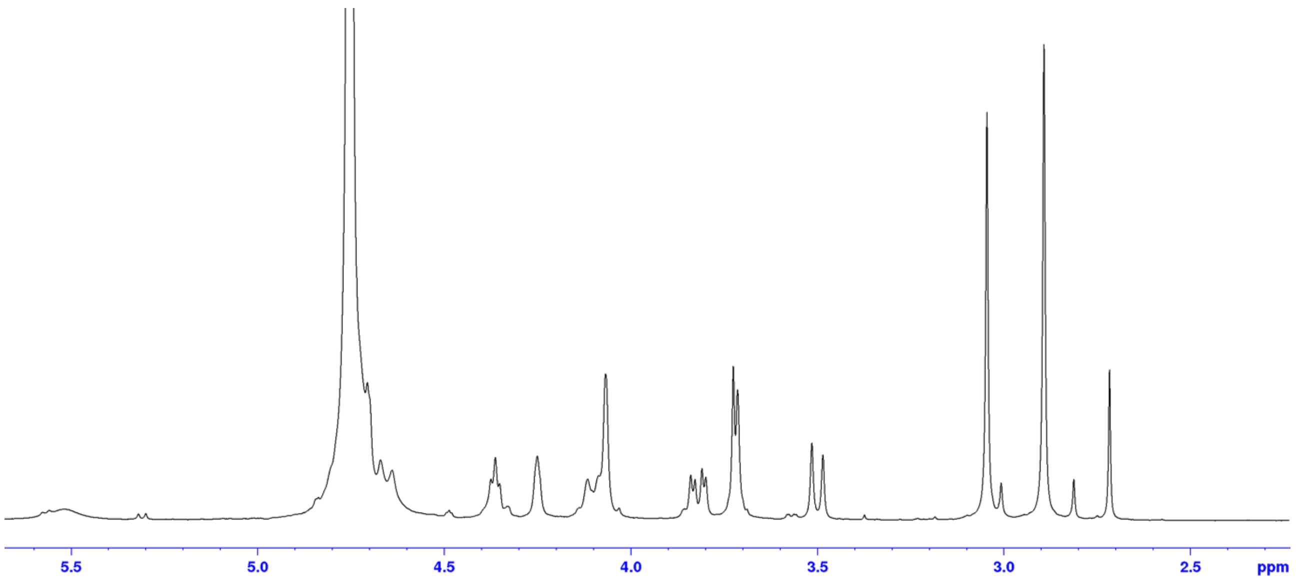
5.5
5.0
4.5
4.0
3.5
3.0
2.5
ppm

b

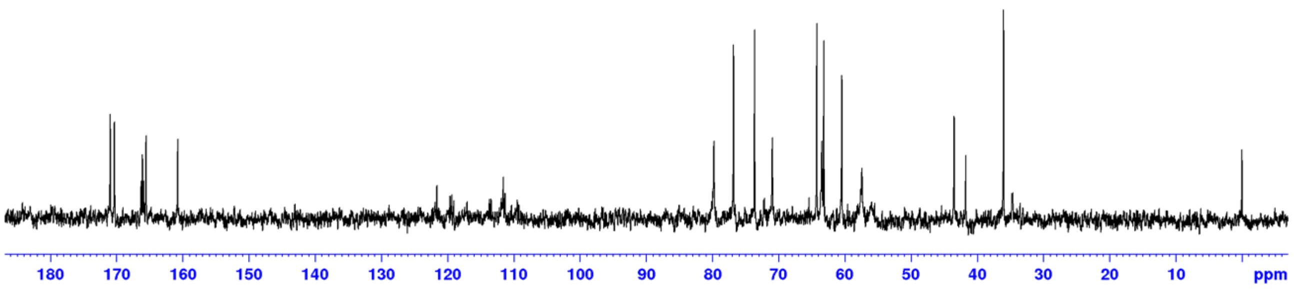
180
170
160
150
140
130
120
110
100
90
80
70
60
50
40
30
20
10
ppm

c

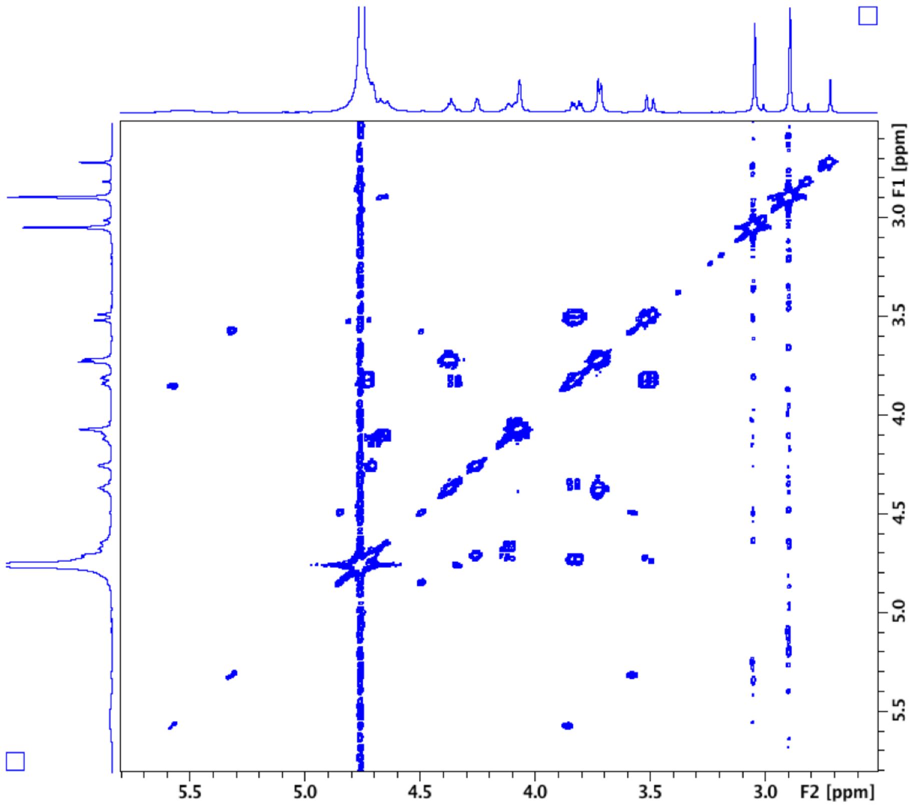

5.5
5.0
4.5
4.0
3.5
3.0
F2 [ppm]
3.0 F1 [ppm]
3.5
4.0
4.5
5.0
5.5

d

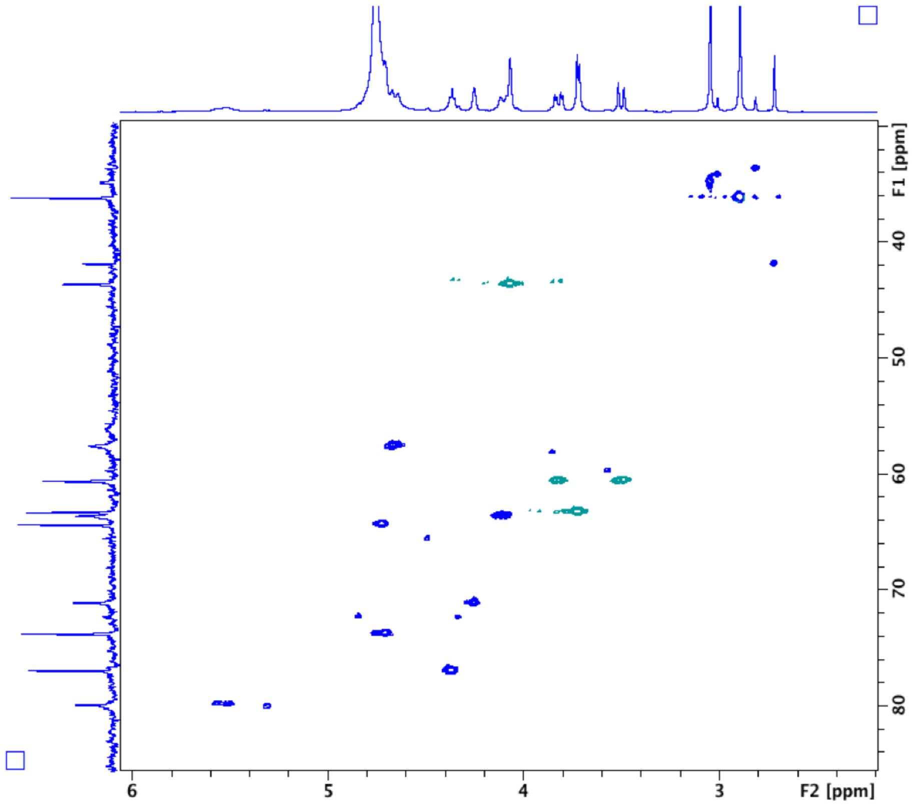

6
5
4
3
F2 [ppm]
F1 [ppm]
40
50
60
70
80

e

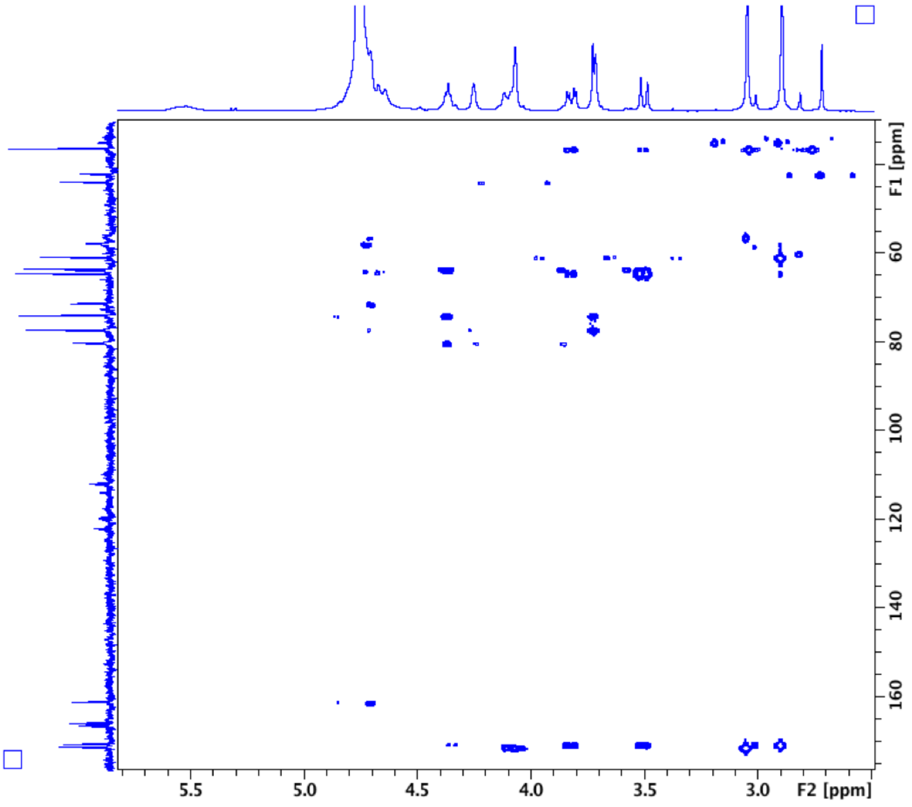

f

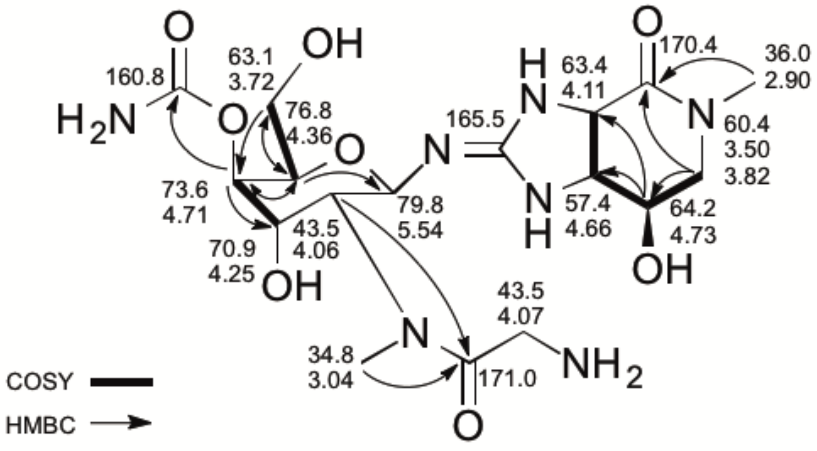

Supplementary Figure 2. Elucidation of the glycinothricin chemical structure. (a) ^{1}H-NMR spectrum of glycinothricin (Fig. 1a, compound **1**). (b) ^{13}C-NMR spectrum **1**. (c) ^{1}H-^{1}H -COSY spectrum of **1**. (d) HMQC-TOCSY spectrum of **1**. (e) HMBC spectrum of **1**. (f) The chemical structure of **1** elucidated by NMR, in which COSY and HMBC are respectively represented as bold bonds and arrows.

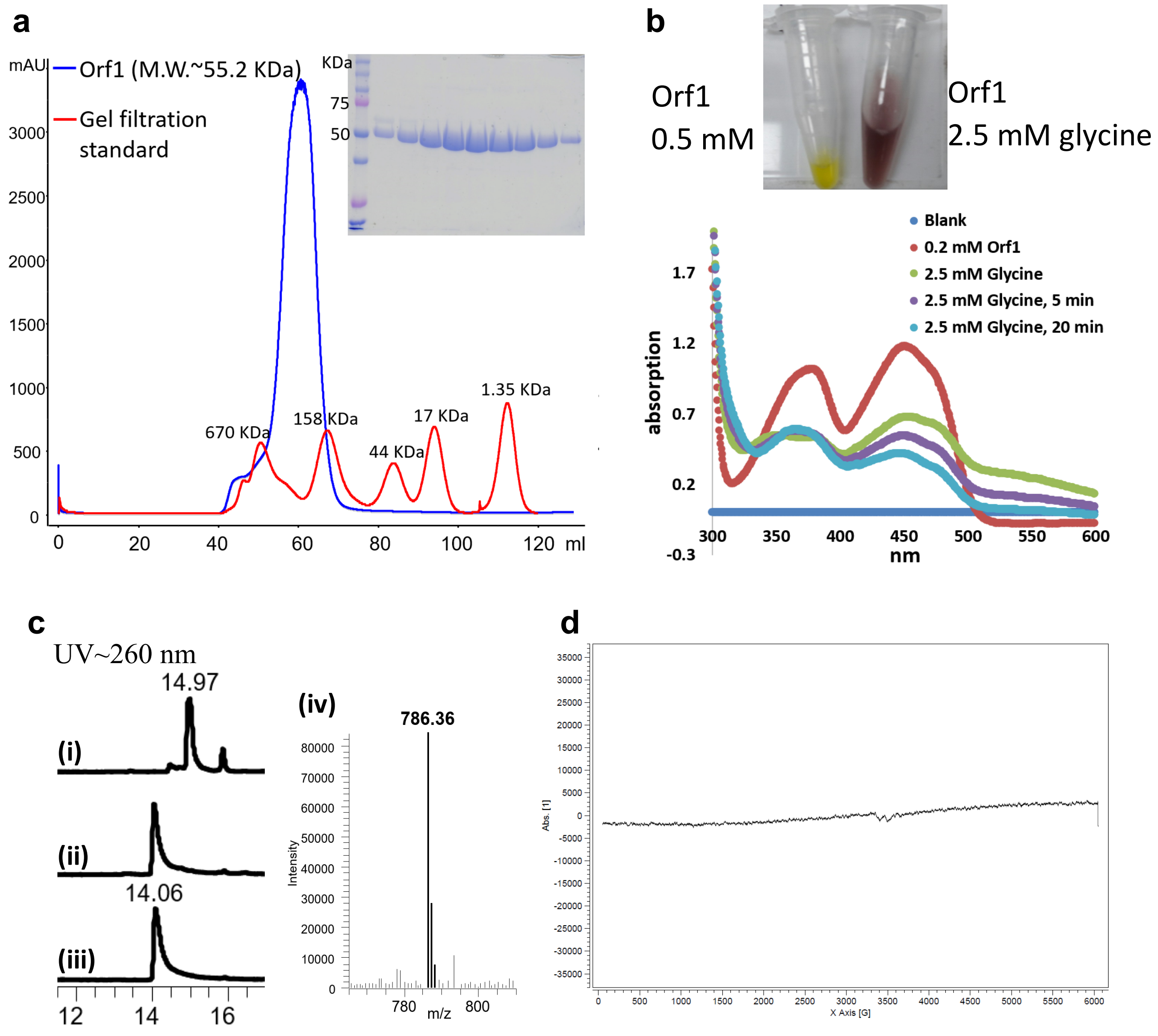

Supplementary Figure 3. FAD-containing Orf1 is tetrameric in solution. (a) The tetrameric Orf1 was estimated to be ~220 kDa using gel filtration chromatography installed with a Hiload 16/60 Superdex 200 pg column. The gel filtration experiments of Orf1 were run with four independent replicates (*n*=4) and the data are provided as a Source Data file. (b) The ultraviolet-visible (UV-Vis) spectrum shows that yellow Orf1 changes to purple-red one when 2.5 mM glycine was added into. (The purple color indicates that the reaction solution is likely a mixture of all possible states of FAD (FAD_{ox} (yellow), half reduced FAD (semiquinone/hydroquinone, blue or red) and FAD_{red} (colorless), particularly C4-peroxide-flavin (purple blue)). (c) The HPLC chromatographs and MS spectrum of: (i) FMN standard, (ii) FAD standard and (iii) the supernatant of denatured Orf1, by subjecting to HPLC analysis equipped with a C18 column (4.6 × 250 mm, 5 μm, Prodigy, Phenomenex), (iv) Mass spectrum of the peak in (iii), confirming FAD is the cofactor of Orf1. (d) X-band EPR analysis for Orf1 in a reaction solution with addition of glycine and **7** was conducted at 77 K.

a

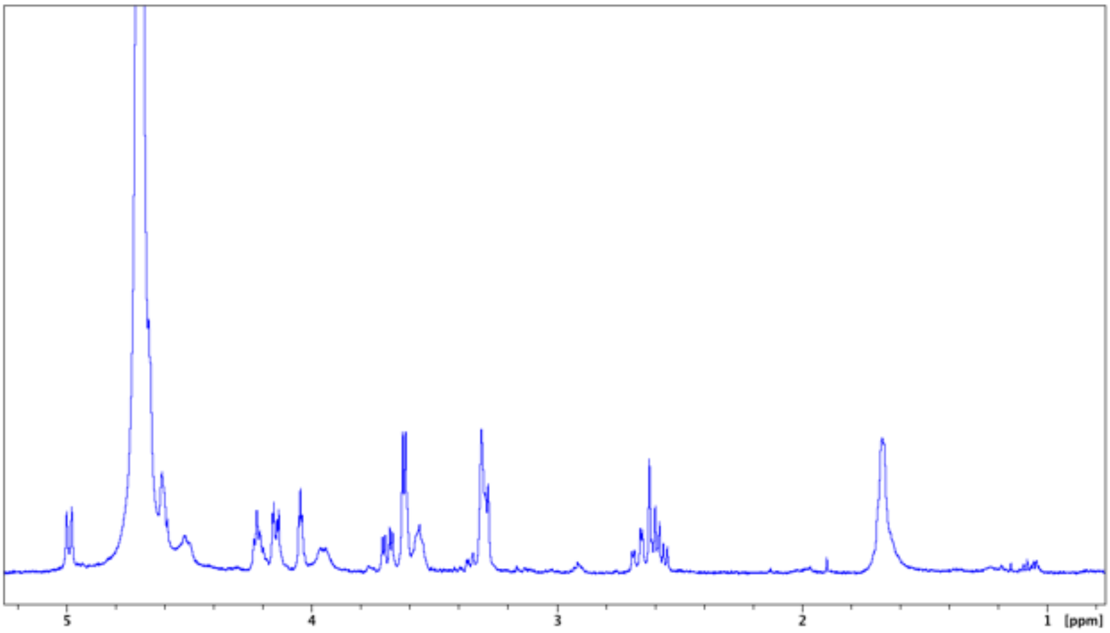

b

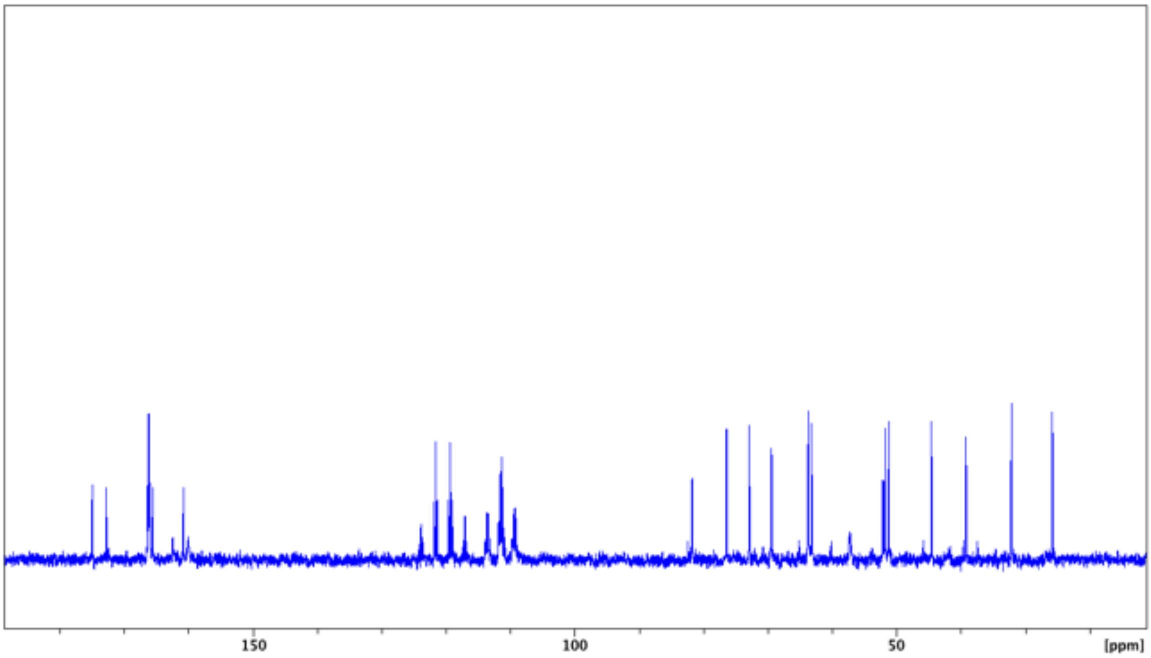

c

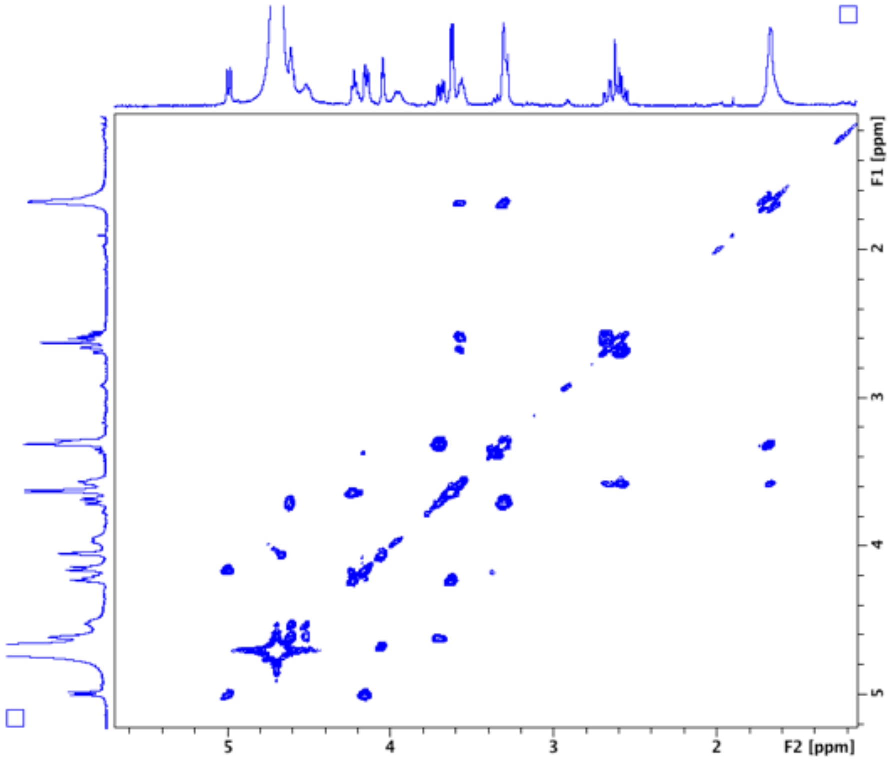

d

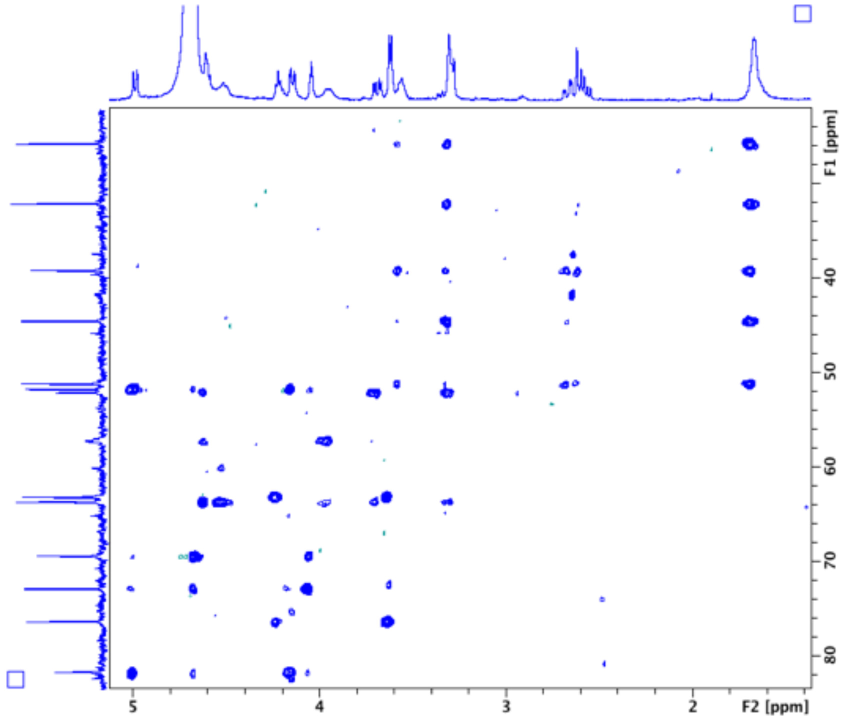

e

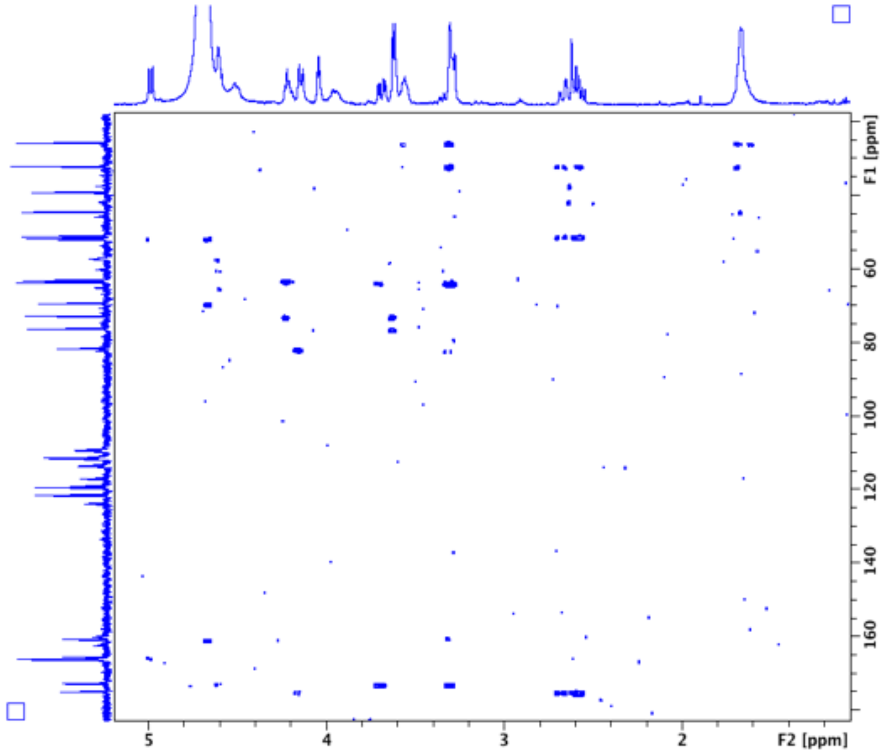

f

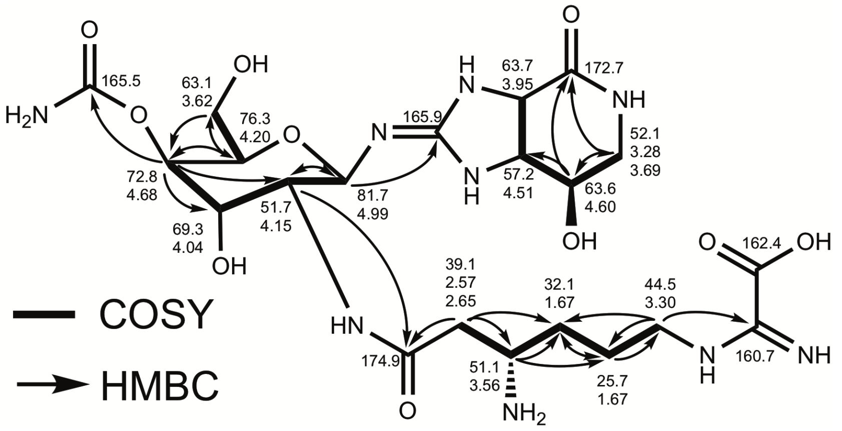

Supplementary Figure 4. Elucidation of the iminoacetyl-ST-F chemical structure. (a) ^{1}H-NMR spectrum of iminoacetyl-ST-F (Fig. 1a, compound **8**). (b) ^{13}C-NMR spectrum **8**. (c) ^{1}H-^{1}H -COSY spectrum of **8**. (d) HMQC-TOCSY spectrum of **8**. (e) HMBC spectrum of **8**. (f) The chemical structure of **8** elucidated by NMR, in which COSY and HMBC are respectively represented as bold bonds and arrows.

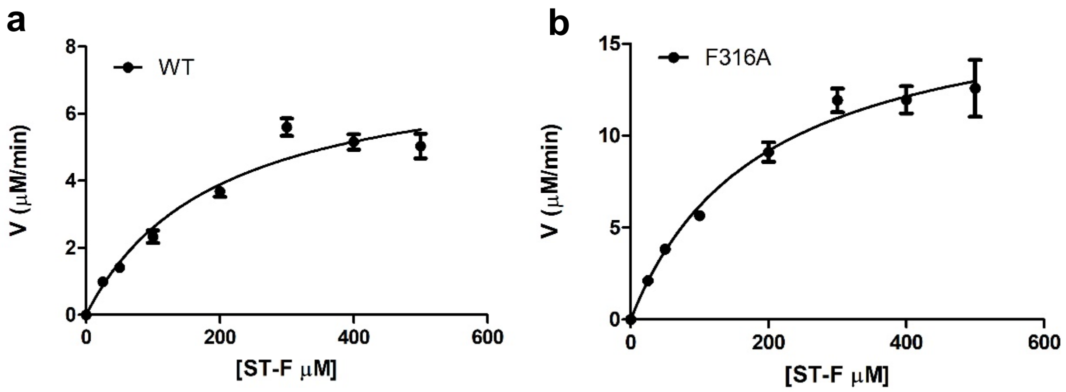

Supplementary Figure 5. Michaelis-Menten kinetics of wild-type and F316A versus ST-F. Enzyme kinetics for iminoacetylation of ST-F catalyzed by wild-type or F316A were estimated in a standard reaction solution (100 mM phosphate buffer at pH 8.0, 2.5 μM WT or F316A enzyme, 2 mM glycine and 25-500 μM ST-F at 37 °C) under the pseudo-first-order condition for a period of 10 minutes. The K_m and k_{cat} determined for WT and F316A are K_m ~196 ± 50.4 μM, k_{cat} ~ 3.077 ± 0.3252 min^{-1} and K_m ~ 190 ± 36.8 μM, k_{cat} ~ 7.16 ± 0.5623 min^{-1}, respectively. The assays of WT and F316A were run with two independent replicates (n=2) and the data were presented as mean values +/- SEM. Source data are provided as a Source Data file.

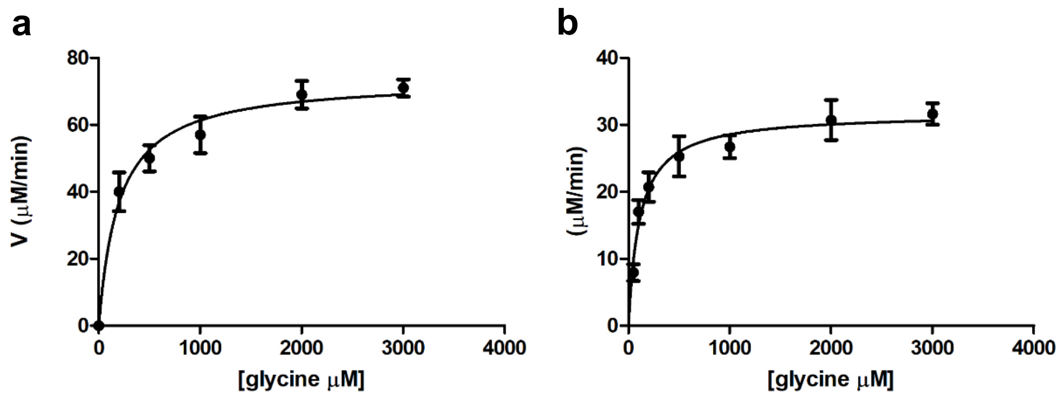

Supplementary Figure 6. Michaelis-Menten kinetics of glycine oxidation catalyzed by Orf1 and ThiO. Enzyme kinetics for glycine oxidation catalyzed by Orf1 or ThiO was estimated in a standard reaction solution (100 mM phosphate buffer at pH 8.0, 200-3000 μM glycine, 100 μM Orf1 or 20 μM ThiO at 37 °C) under the pseudo-first-order condition within 1 minute. (a) The basic kinetic parameters for Orf1 are: K_m ~201.5 ± 50.0 μM and k_{cat} ~ 0.7367 ± 0.0355 min^{-1}. (b) The basic kinetic parameters for ThiO are: K_m ~ 111.8 ± 21.6 μM and k_{cat} ~ 1.587 ± 0.0675 min^{-1}. The assays of Orf1 and ThiO were run with two independent replicates (*n*=2) and the data were presented as mean values +/- SEM. Source data are provided as a Source Data file.

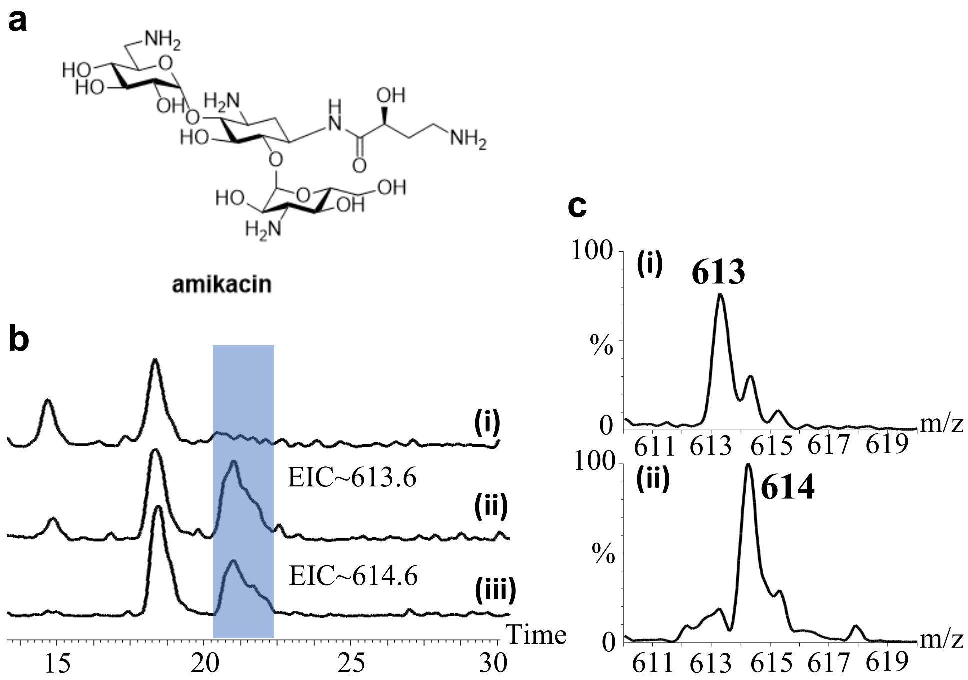

Supplementary Figure 7. Orf1-mediated reactions in the presence of amikacin and glycine. (a) Chemical structure of amikacin. (b) The EIC traces for the formation of formimidoyl-amikacin (*m*/*z* 613.6 $[M+H]^+$) in the condition of (i) denatured Orf1 with glycine, (ii) Orf1 with glycine (*m*/*z* 613.8 $[M+H]^+$)), (iii) Orf1 with ^{15}N-glycine (*m*/*z* 614.8$[M+H]^+$); (c) Mass spectra of the products in the condition of (i) Orf1 with glycine, and (ii) Orf1 with ^{15}N-glycine. Source data are provided as a Source Data file.

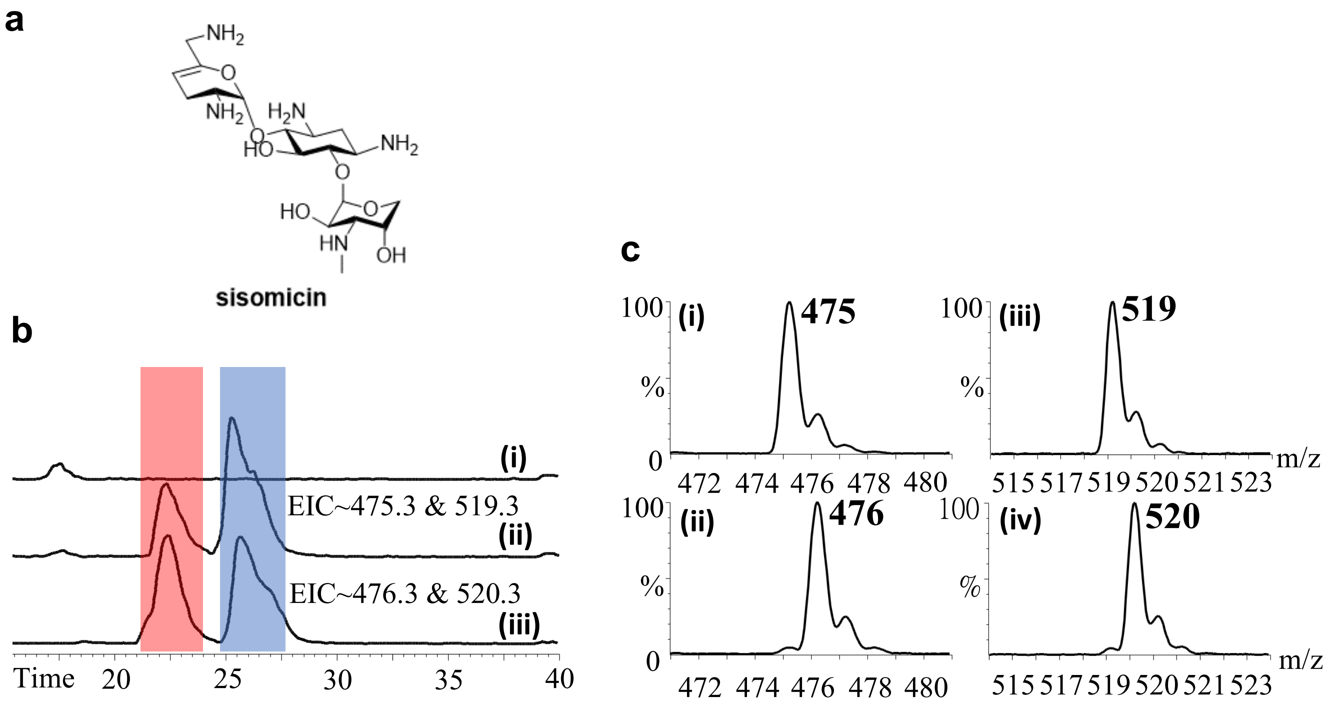

Supplementary Figure 8. Orf1-mediated reactions in the presence of sisomicin and glycine. (a) Chemical structure of sisomicin. (b) The EIC traces for the formation of formimidoyl-sisomicin (*m/z* 475.3 $[M+H]^+$, highlighted with blue background) and iminoacetyl-sisomicin (*m/z* 519.3 $[M+H]^+$, highlighted with red background) in the reaction conditions of (i) denatured Orf1 with glycine, (ii) Orf1 with glycine, (iii) Orf1 with ^{15}N-glycine; (c) Mass spectra of formimidoyl-sisomicin in the reaction conditions of (i) Orf1 with glycine (*m/z* 475 $[M+H]^+$), and (ii) Orf1 with ^{15}N-glycine (*m/z* 476 $[M+H]^+$). Mass spectra of iminoacetyl-sisomicin in the reaction conditions of (iii) Orf1 with glycine (*m/z* 519 $[M+H]^+$), and (iv) Orf1 with ^{15}N-glycine (*m/z* 520 $[M+H]^+$). Source data are provided as a Source Data file.

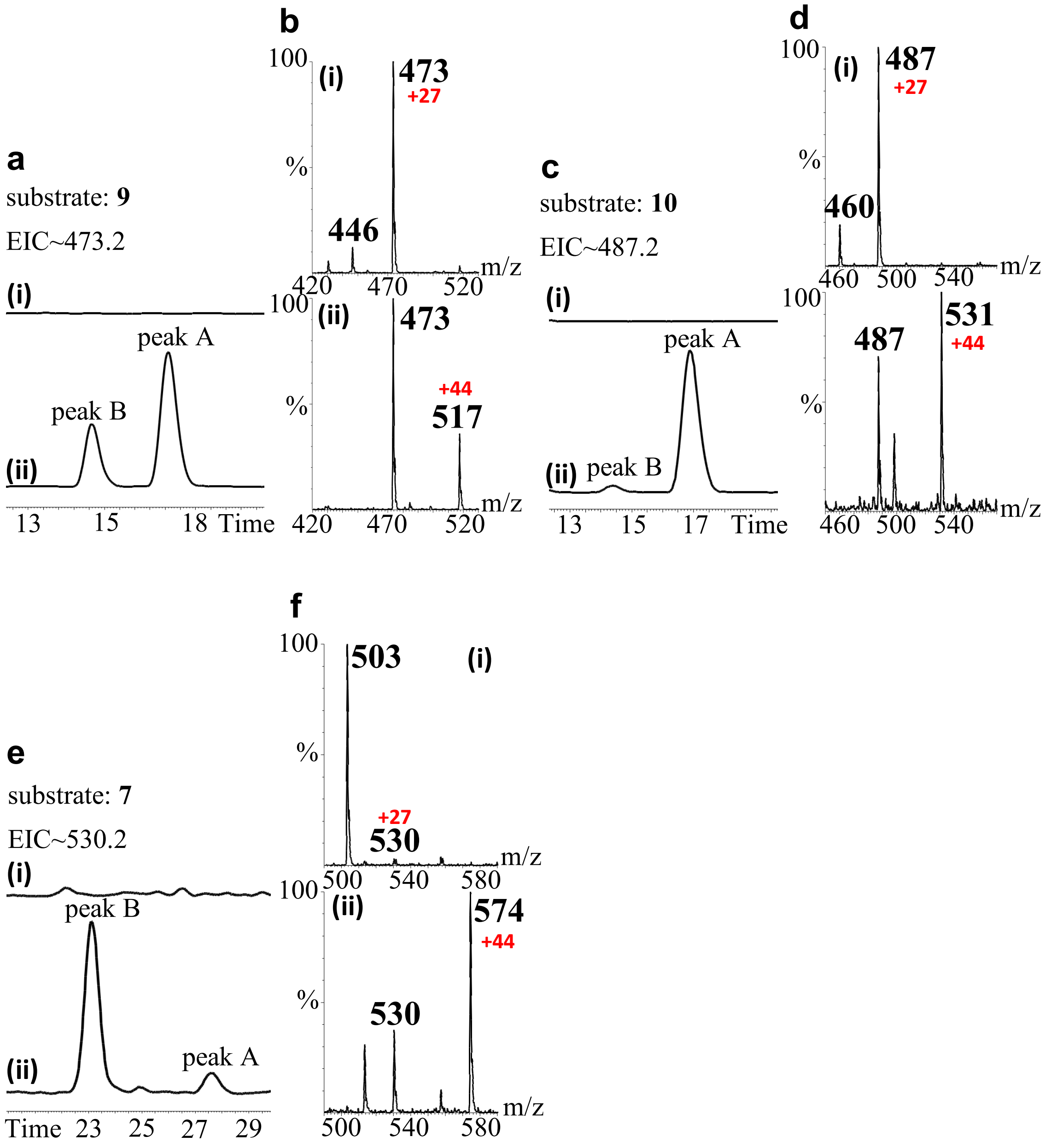

Supplementary Figure 9. Orf1-mediated reactions in the presence of 3-aminopropionylthricin, 4-aminobutylthricin and ST-F. (a) The EIC traces of formimidoylated **9** (*m/z* 473.2 $[M+H]^+$) from the reaction conditions of (i) denatured Orf1 with glycine, (ii) Orf1 with glycine. (b) Mass spectra of the products from the Orf1-mediated reactions: (i) peak A formimidoylated **9**, (ii) peak B iminoacetylated **9**. (c) The EIC traces of formimidoylated **10** (*m/z* 487.2 $[M+H]^+$) from the reaction conditions of (i) denatured Orf1 with glycine, (ii) Orf1 with glycine. (d) Mass spectra of the products from the Orf1-mediated reactions: (i) peak A formimidoylated **10**, (ii) peak B iminoacetylated **10**. (d) The EIC traces of *N*-formimidoylated **7** (*m/z* 530.2 $[M+H]^+$) from the reaction conditions of (i) denatured Orf1 with glycine, (ii) Orf1 with glycine. (e) Mass spectra of the products from the Orf1-mediated reactions: (i) peak A *N*-formimidoylated **7**, (ii) peak B **8**. Source data are provided as a Source Data file.

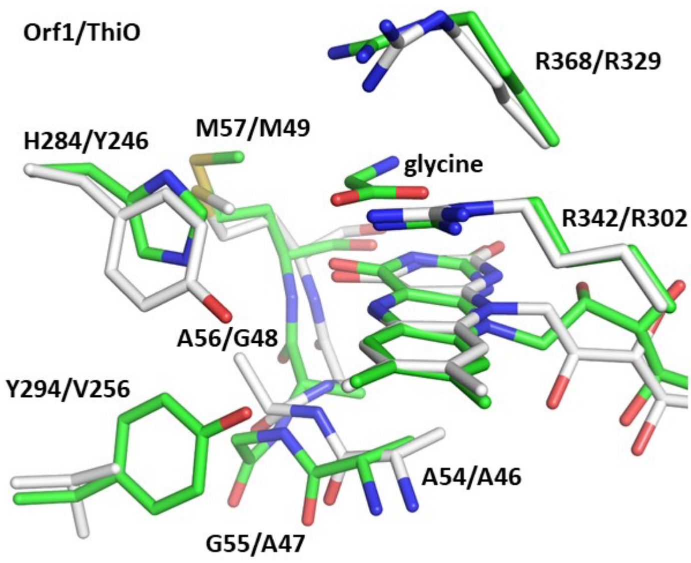

Supplementary Figure 10. Superposition of two glycine binding sites from Orf1 and ThiO. The glycine binding sites of Orf1-glycine and ThiO (pdb: 1NG3) are colored gray and green, respectively. Arg368 and R342 intimately interact with glycine. There are subtle differences at the isoalloxazine ring, H284/Y246, Y294/V256, A56/G48 and G55/A47 between the two binding sites.

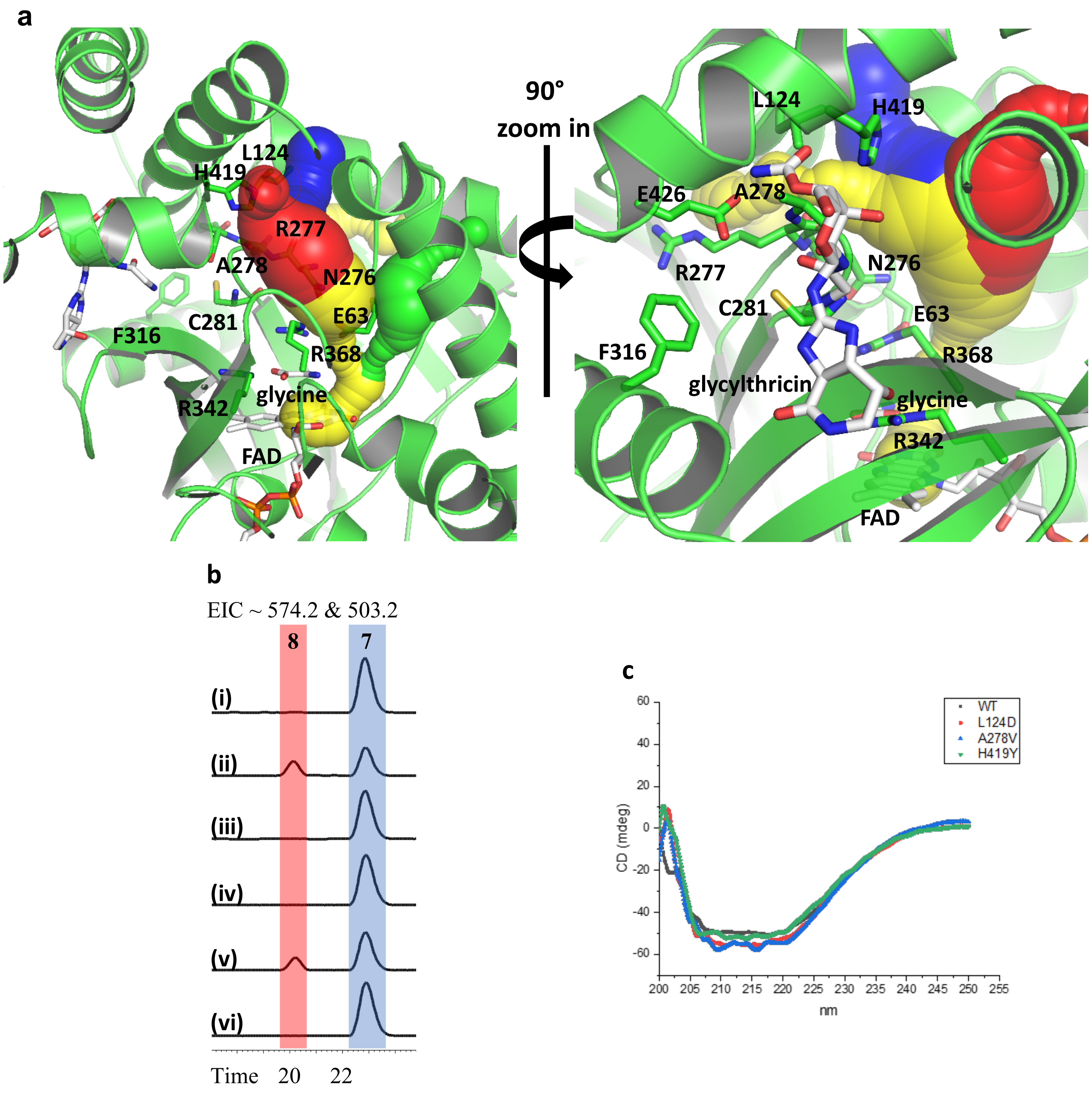

Supplementary Figure 11. Identification of tunnels involved in glycine/intermediate transport in Orf1. (a) Four possible tunnels in proximity to the glycine/FAD binding site for glycine/intermediate transport were predicted using CAVER 3.0. Only one tunnel colored yellow is a candidate for oxime or possible reactive species channeled to the second reaction site (close to C281 and E426) likely as a result of the geolocation and biochemical necessity (see below). The other three are converged through a bottleneck to the FAD/glycine binding site, whereby it may serve as a checking point gating the entrance of glycine/oxygen. The glycylthricin binding site comprising Cys281 is at the other end of the FAD/glycine binding site separated by β-strands including β11-β15. This spatial arrangement expedites the reaction along the assembly line in an orderly and controllable manner. (b) The EIC traces of **7** (m/z 503.2 $[M+H]^+$) and **8** (m/z 574.2 $[M+H]^+$) for reactions catalyzed by (i) denatured Orf1, (ii) Orf1, (iii) L124D, (iv) A278V, (v) A280S, (vi) H419Y. The residues that constitute the tunnel were individually mutated and subjected to biochemical examinations: L124D, A278V and H419Y are unable to convert **7** to **8** but, nevertheless, they

still maintain 74.7%, 84.2% and 91.1% of the glycine oxidation activity, respectively. (c) Circular dichroism spectra of WT, L124D, A278V and H419Y were recorded for 10 μM protein in 100 mM sodium phosphate pH 7.0 on a Jasco J-815 spectropolarimeter. The CD spectrometric analysis suggests that these mutations do not provoke significant structural changes, on which the glycine oxidation activity functions normally but the intermediate delivering is terminated. Source data are provided as a Source Data file.

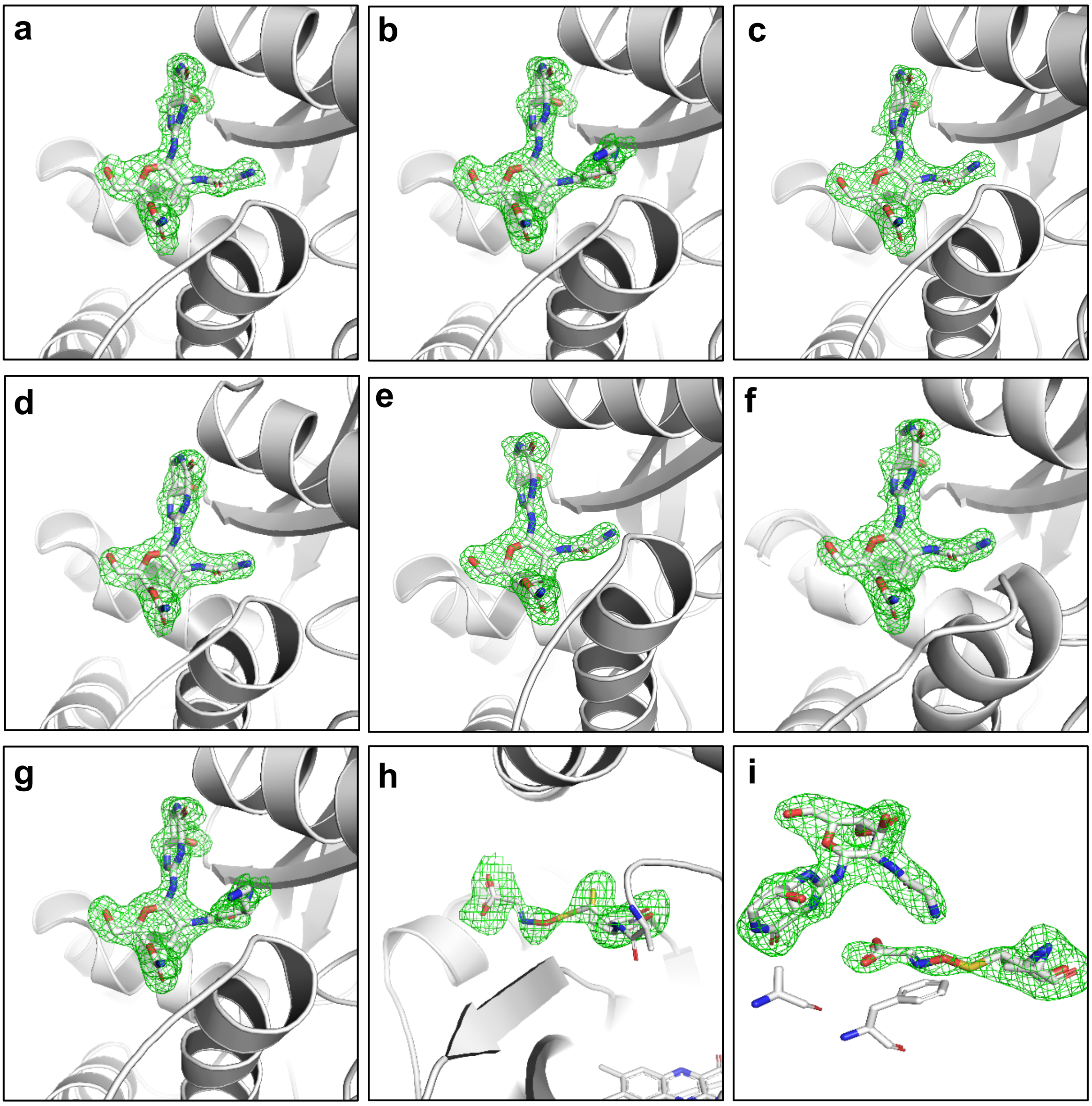

Supplementary Figure 12. The unbiased F_o-F_c omit maps for ligands identified in the crystal structures determined in this study. (a) Orf1-glycylthricin (B chain), (b) Orf1-4-aminobutylthricin (E chain), (c) C281S-glycylthricin (B chain), (d) R342A-glycylthricin (A chain), (e) E426Q-glycylthricin (B chain), (f) F316A-glycylthricin (B chain), (g) F316A-ST-F (B chain), (h) Orf1-iminoglycine adduct (H chain) and (i) E312A-glycylthricin-iminoglycine adduct (A chain). The F_o-F_c omit difference density is contoured at 3 σ and 2.5 σ for a-f, i and g-h, respectively.

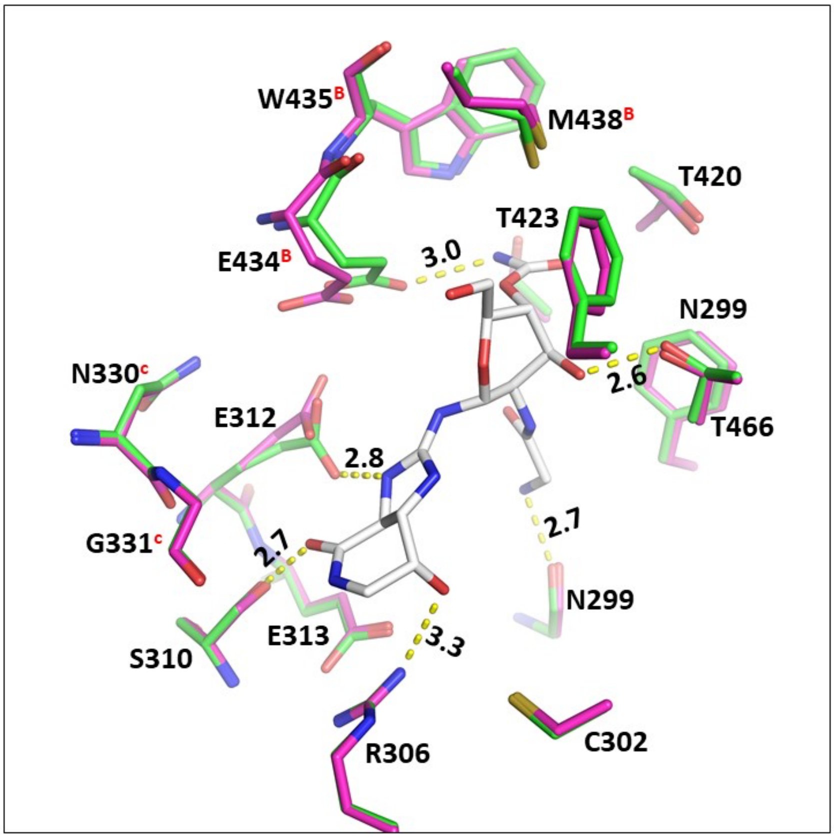

Supplementary Figure 13. Superposition of the crystal structures of Orf1-apo and Orf1-glycine-glycylthricin. The residues of the apo and glycylthricin-liganded structures are colored magentas and green, respectively. E434 (B subunit) and E312 undergo substrate-induced conformational changes. The distance between two atoms is indicated with a yellow dashed line.

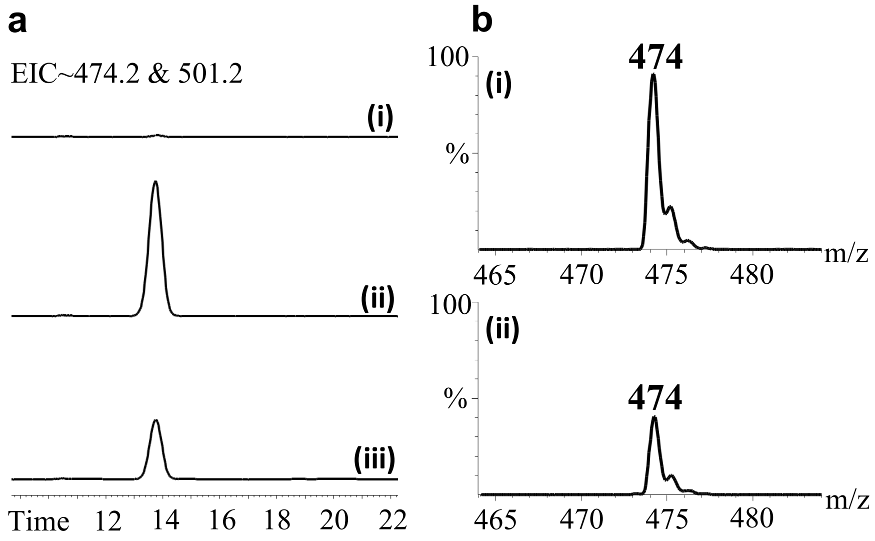

Supplementary Figure 14. *In vitro* assay of Orf1 and SttE with glycylthricin. (a) The EIC traces for the acetylation reactions catalyzed by SttE in the presence of **5** or **6** in a reaction solution containing: (i) Orf1 + glycine and denatured SttE + acetyl-CoA, (ii) denatured Orf1 + glycine and SttE + acetyl-CoA, (iii) Orf1 + glycine and SttE + acetyl-CoA (acetylated **4** or **5** if formed would have a mass of *m*/*z* 474.2 $[M+H]^+$ or *m*/*z* 501.2 $[M+H]^+$, respectively). (b) Mass spectra of the products from the Orf1 and SttE-coupled reactions: (i) acetylated **4** was detected in a reaction added with denatured Orf1 + glycine and SttE + acetyl-CoA, (ii) only was acetylated **4** but not acetylated **5** detected in a reaction added with Orf1 + glycine and SttE + acetyl-CoA. The absence of acetylated **5** indicated that **5** is not a substrate of SttE. Each reaction was initiated with addition of 2.5 μM Orf1 into a solution containing 0.1 mM **4**, 1 mM glycine, 100 mM sodium phosphate pH 8 and incubated at 37°C for 1 hr. For the acetylation reaction, 25 μM SttE and 2 mM acetyl-CoA were added into the reaction solutions and incubated at 37°C for another 30 mins. Source data are provided as a Source Data file.

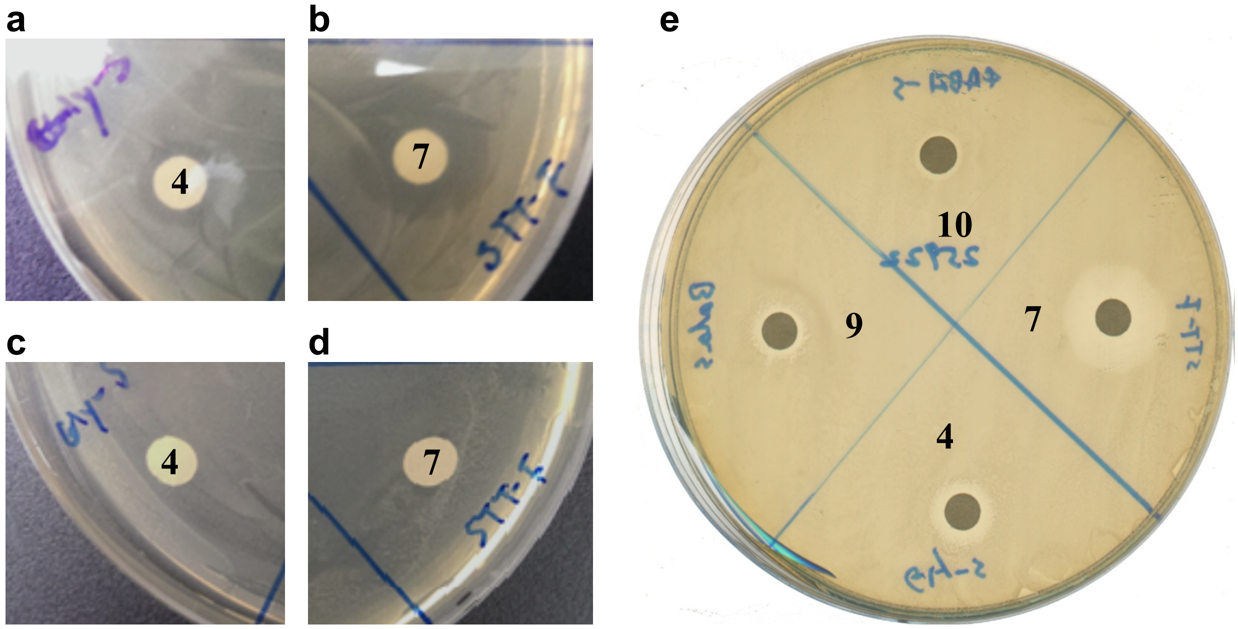

Supplementary Figure 15. Disc diffusion assays for compounds 4, 7, 9 and 10. (a, b) Compounds **4** and **7** were respectively added in paper discs and placed on bacterial lawns of *E. coli* BL21 (DE3) harboring pET28a empty vectors. (c, d) Compounds **4** and **7** were respectively added in paper discs and placed on bacterial lawns of *E. coli* BL21 (DE3) harboring pET28a-*sttE* vectors. (e) Compounds **4**, **7**, **9** and **10** were added in paper discs on bacterial lawns of the *E. coli* ATCC25922 strain. Source data are provided as a Source Data file.

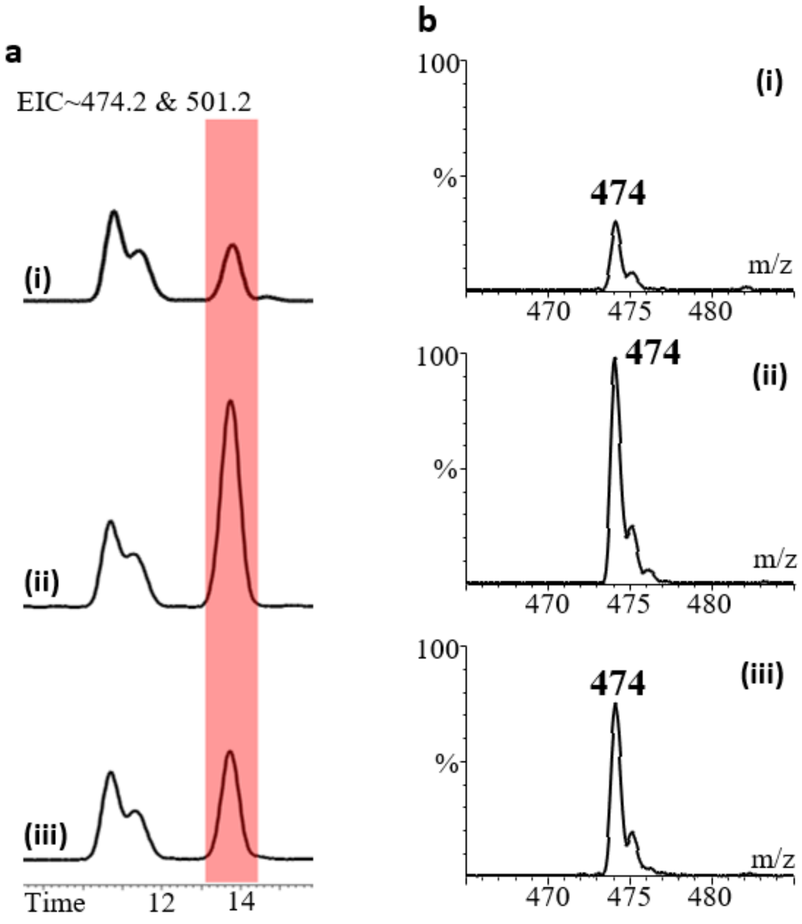

Supplementary Figure 16. *In vitro* assay of Orf1 and AAC(6')-Ie-APH(2'')-Ia with glycylthricin. (a) The EIC traces for the acetylation reactions catalyzed by AAC(6')-Ie-APH(2'')-Ia in a reaction solution containing: (i) Orf1 + glycine and denatured AAC(6')-Ie-APH(2'')-Ia + acetyl-CoA, (ii) denatured Orf1 + glycine and AAC(6')-Ie-APH(2'')-Ia + acetyl-CoA, (iii) Orf1 + glycine and AAC(6')-Ie/APH(2'') + acetyl-CoA (acetylated **4** or **5** if formed would have a mass of *m/z* 474.2 $[M+H]^+$ or *m/z* 501.2 $[M+H]^+$, respectively). (b) Mass spectra of the products were highlighted with a red background in figure (a). Each reaction was initiated by adding 2.5 μM Orf1 into a solution containing 0.1 mM **4**, 1 mM glycine, 100 mM sodium phosphate pH 8 and incubated at 37°C for 1 hr. For the acetylation reaction, 25 μM AAC(6')-Ie/APH(2'') and 2 mM acetyl-CoA were added to the reaction solutions and incubated at 37°C overnight. If acetylated **4** or **5** were formed, each would have a mass unit of *m/z* 474.2 $[M+H]^+$ or *m/z* 501.2 $[M+H]^+$, respectively. The results reveal that **4** is vulnerable to acetylation modification by AAC(6')-Ie-APH(2'')-Ia while **5** is unsusceptible to the modification. Source data are provided as a Source Data file.

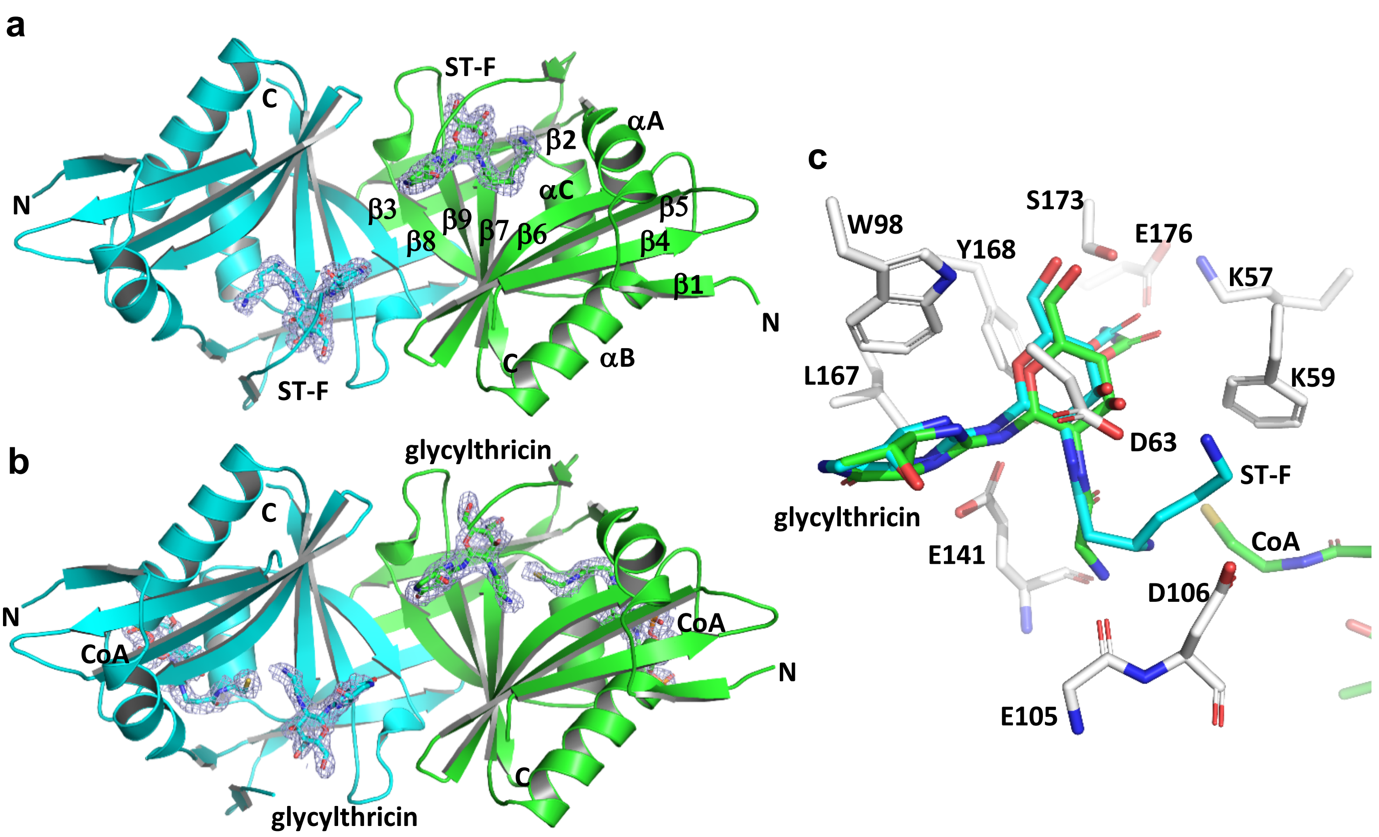

Supplementary Figure 17. Crystal complexes of SttE-ST-F and SttE-CoA-glycylthricin. (a) SttE is a dimer in solution; each protomer contains an acceptor **7** binding site. The 2F$_o$-F$_c$ density map of ligand **7** is contoured at 1 σ in blue mesh . (b) The ternary complex of SttE was determined, where each polypeptide is liganded with an acceptor **4** and a donor (CoA). The 2F$_o$-F$_c$ density map of the ligands is contoured at 1 σ in blue mesh. (c) Superposition of the two complexes shows that the **4** and **7** are well aligned in SttE. The binding-site residues of **4, 4**-CoA and **7** are colored gray, green and cyan, respectively.

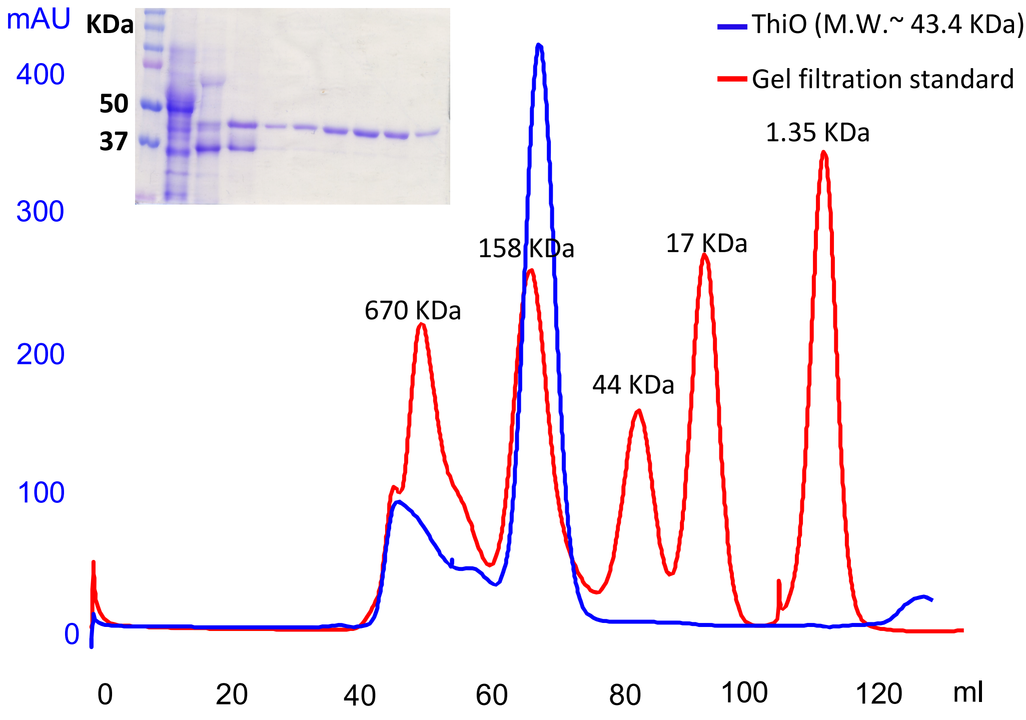

Supplementary Figure 18. Gel filtration chromatograph of ThiO. Tetrameric ThiO was estimated by using gel filtration chromatography installed with a Hiload 16/60 Superdex 200 pg column. The gel filtration experiments were run with three independent replicates (*n*=3) and the data are provided as a Source Data file.

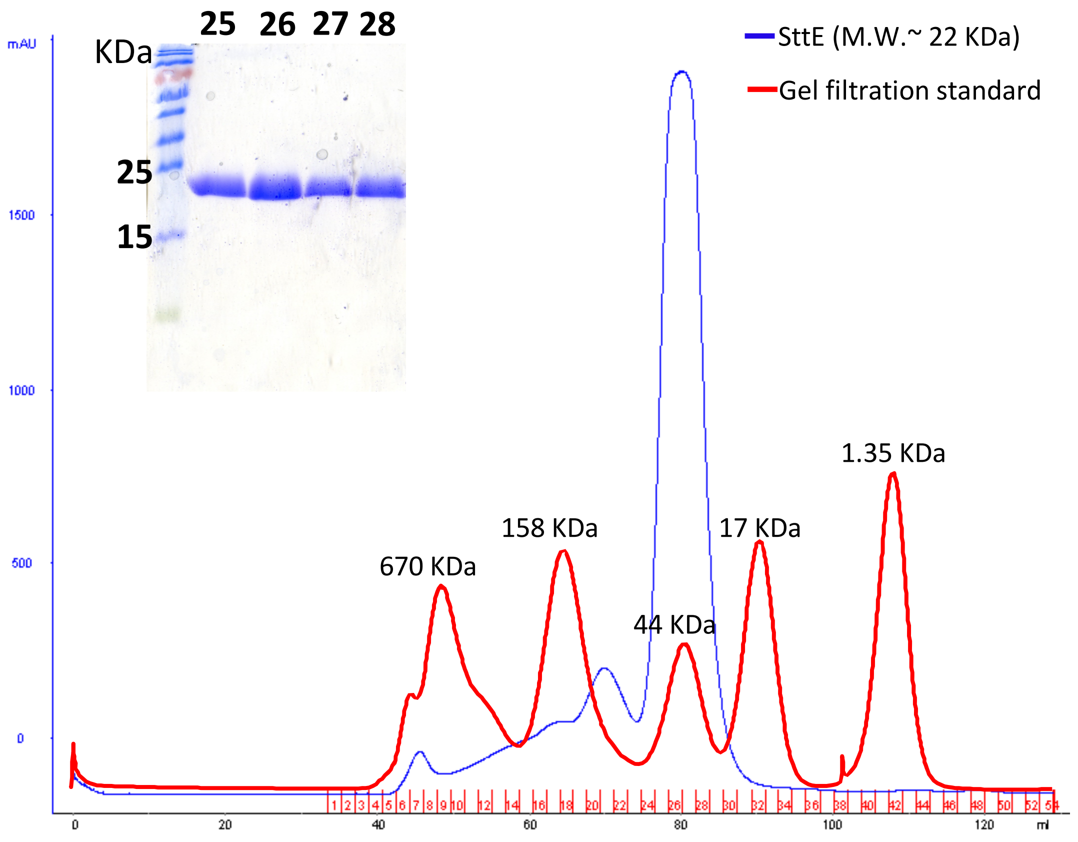

Supplementary Figure 19. Gel filtration chromatograph of SttE. Dimeric SttE was estimated by using gel filtration chromatography installed with a Hiload 16/60 Superdex 200 pg column. The gel filtration experiments were run with three independent replicates (*n*=3) and the data are provided as a Source Data file.

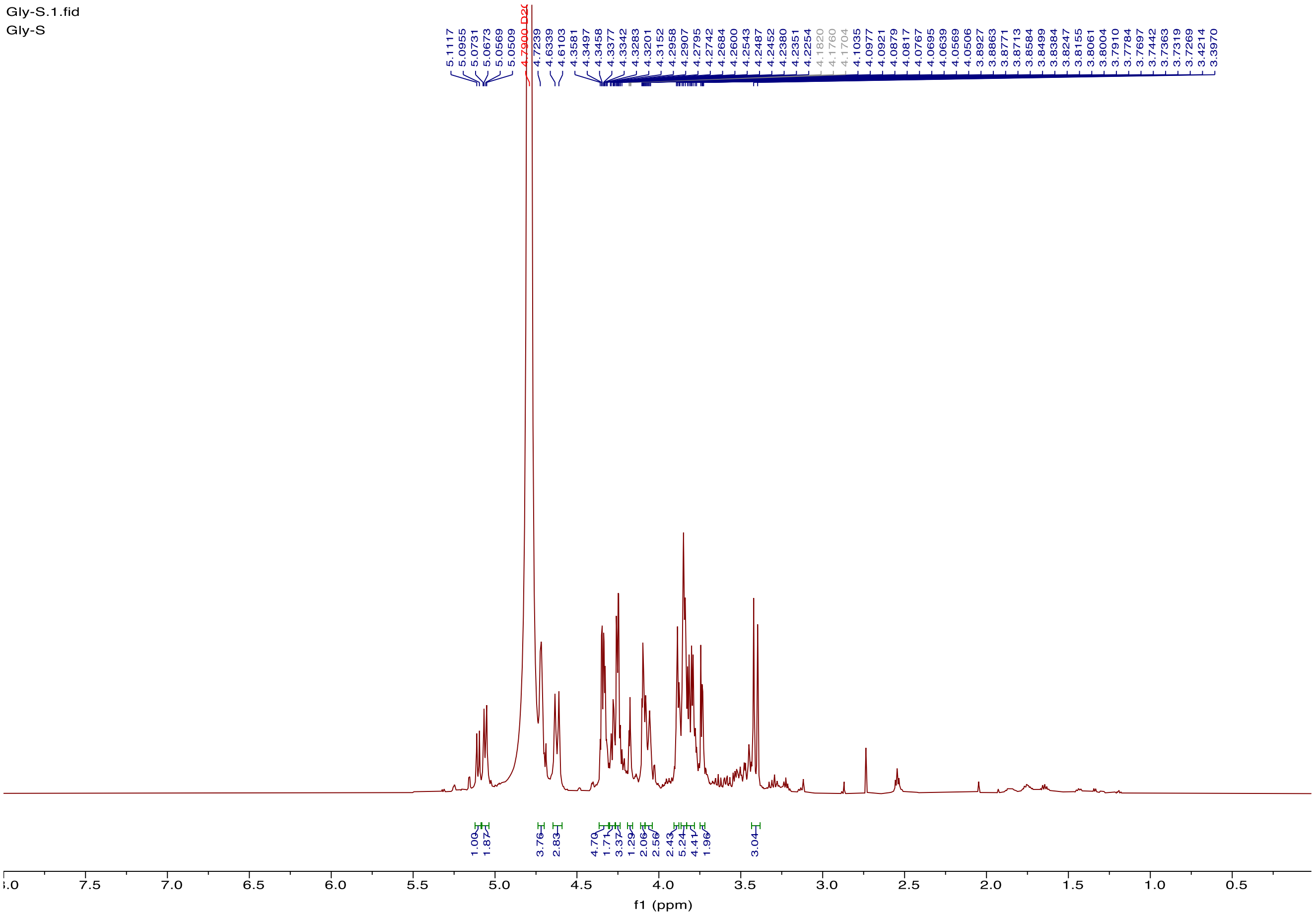

Supplementary Figure 20. ^{1}H NMR of glycylthricin in D_2O (600 MHz)

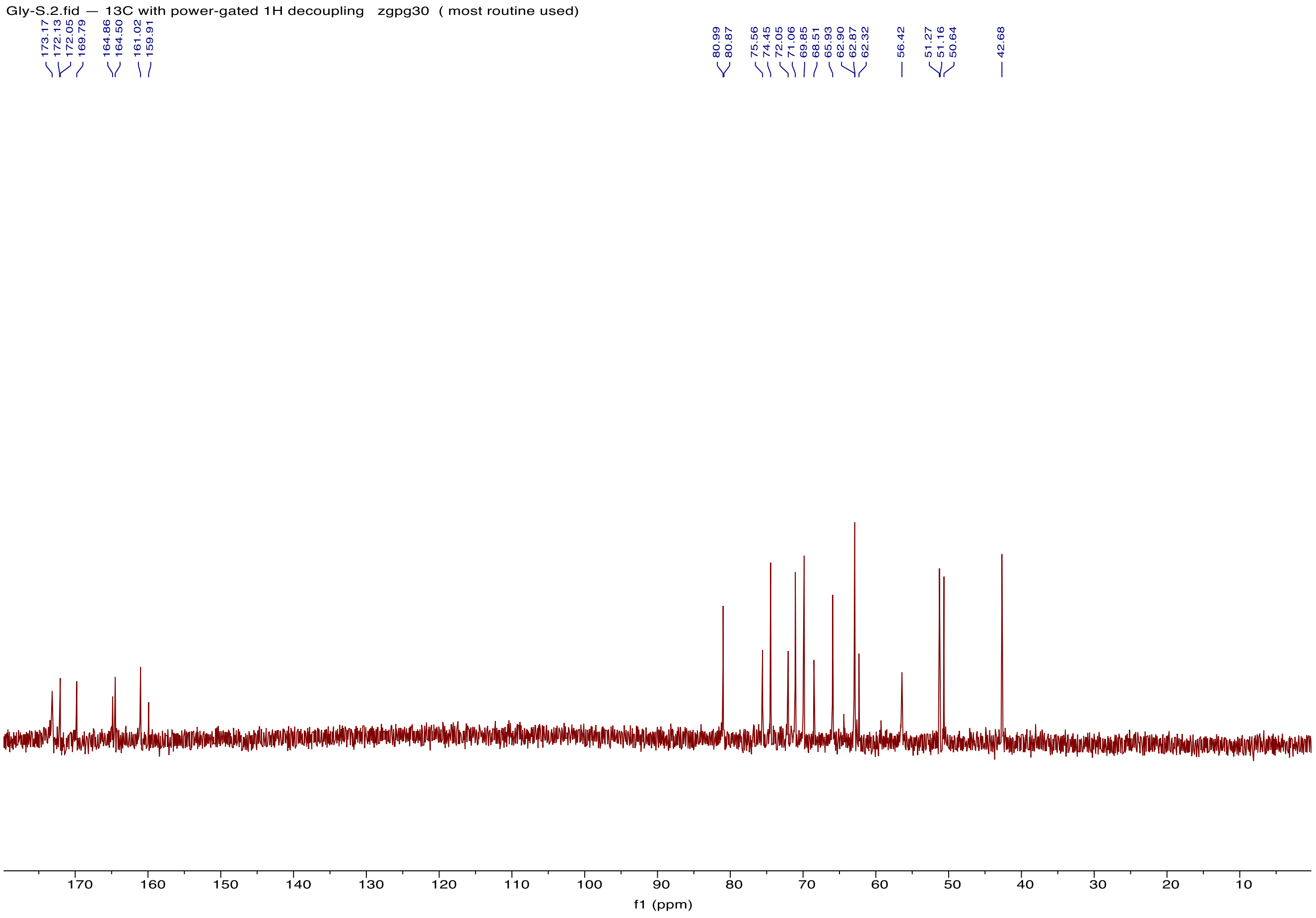

Supplementary Figure 21. ^{13}C NMR of glycylthricin in D_2O (150 MHz)

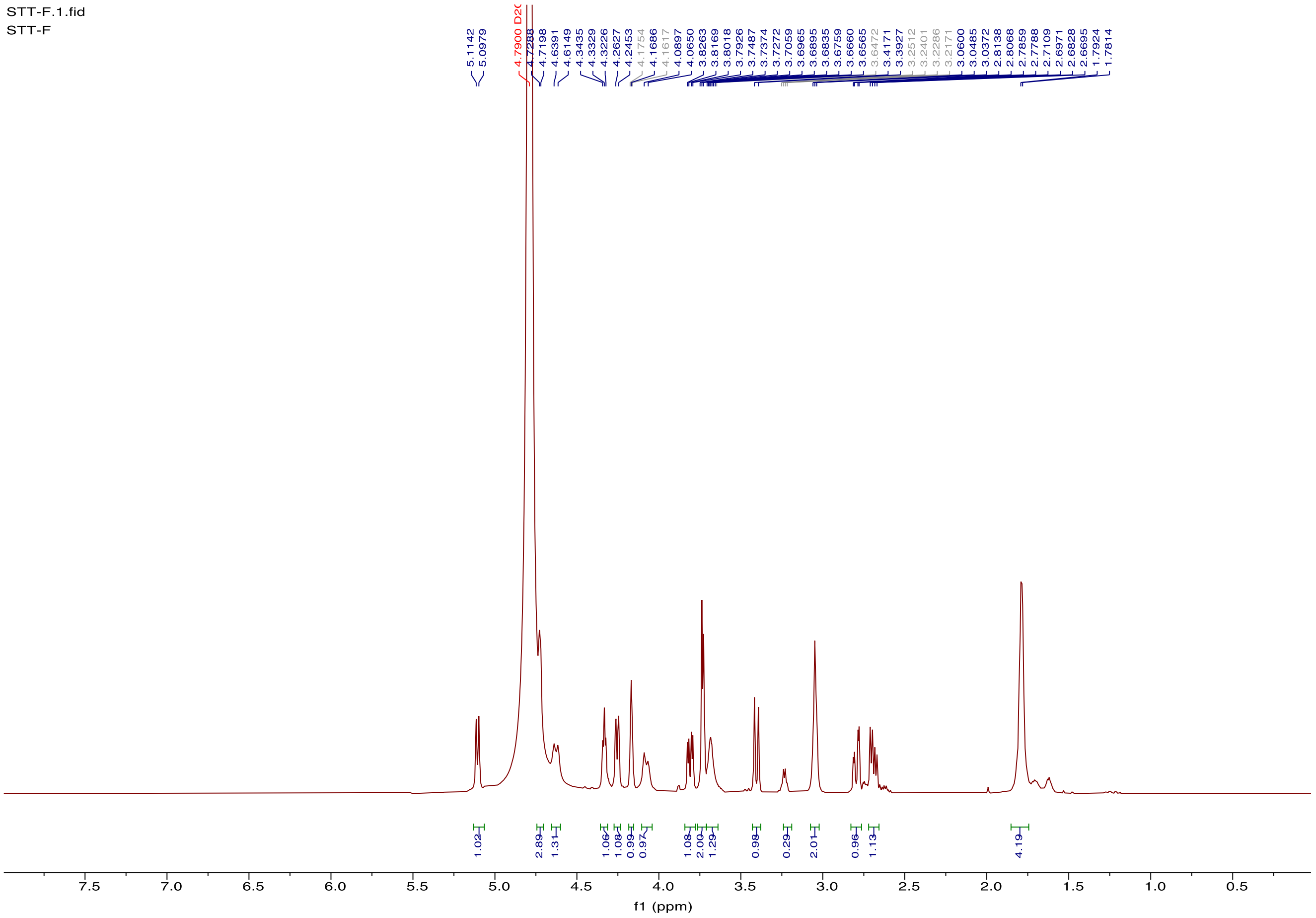

Supplementary Figure 22. ^{1}H NMR of ST-F in D_2O (600 MHz)

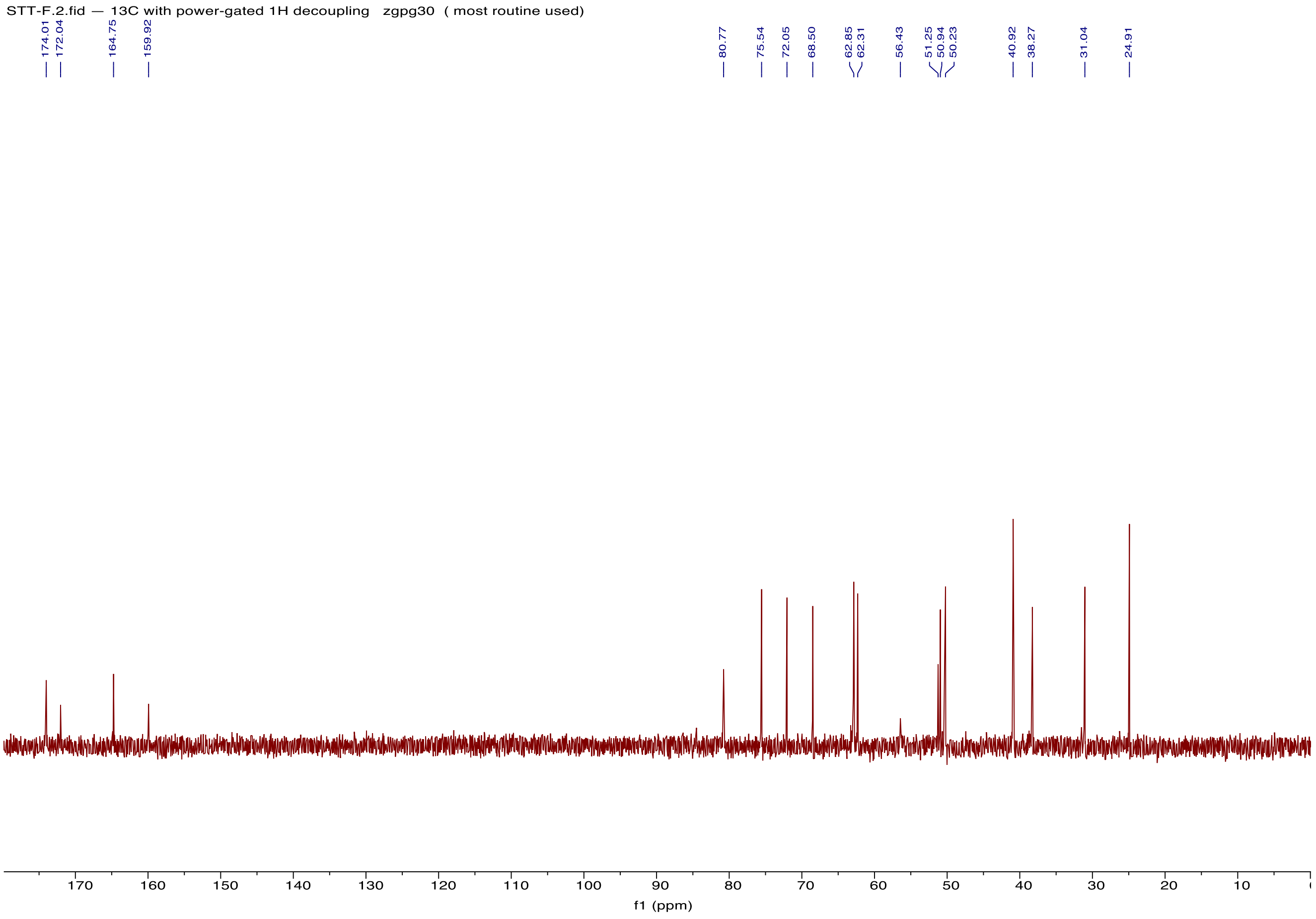

Supplementary Figure 23. ^{13}C NMR of ST-F in D_2O (150 MHz)

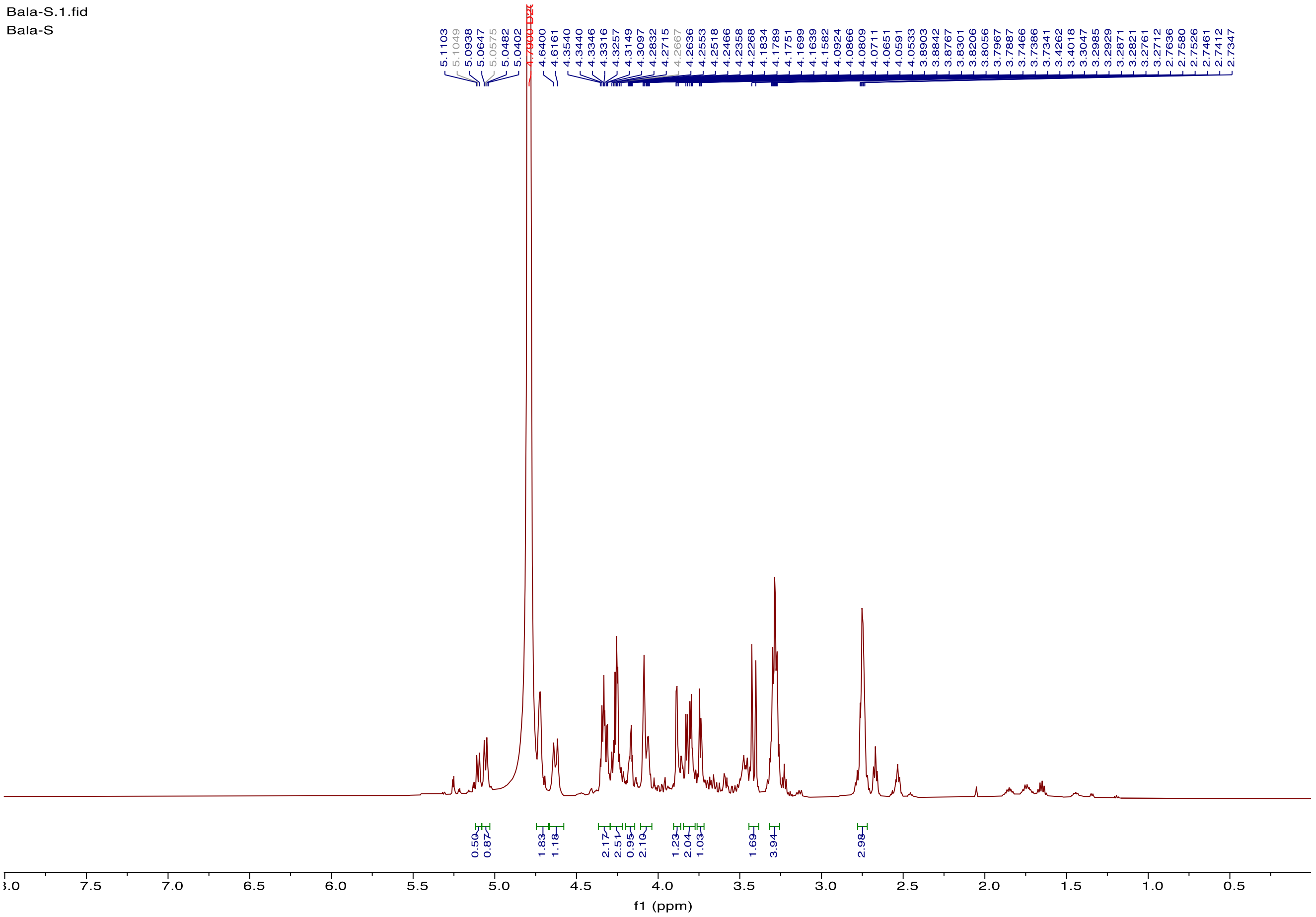

Supplementary Figure 24. ^{1}H NMR of 3-aminopropionylthricin in D_2O (600 MHz)

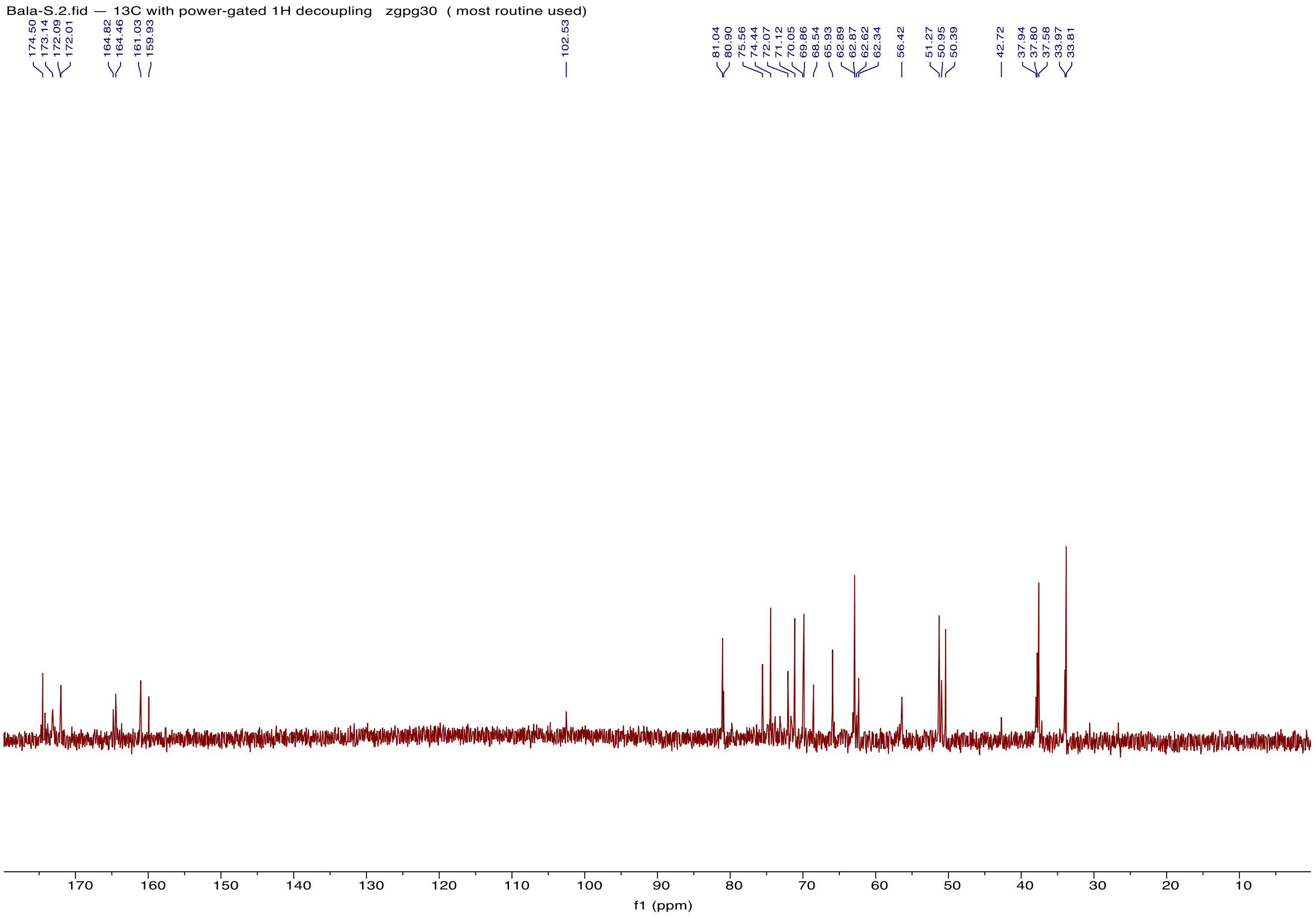

Supplementary Figure 25. ^{13}C NMR of 3-aminopropionylthricin in D_2O (150 MHz)

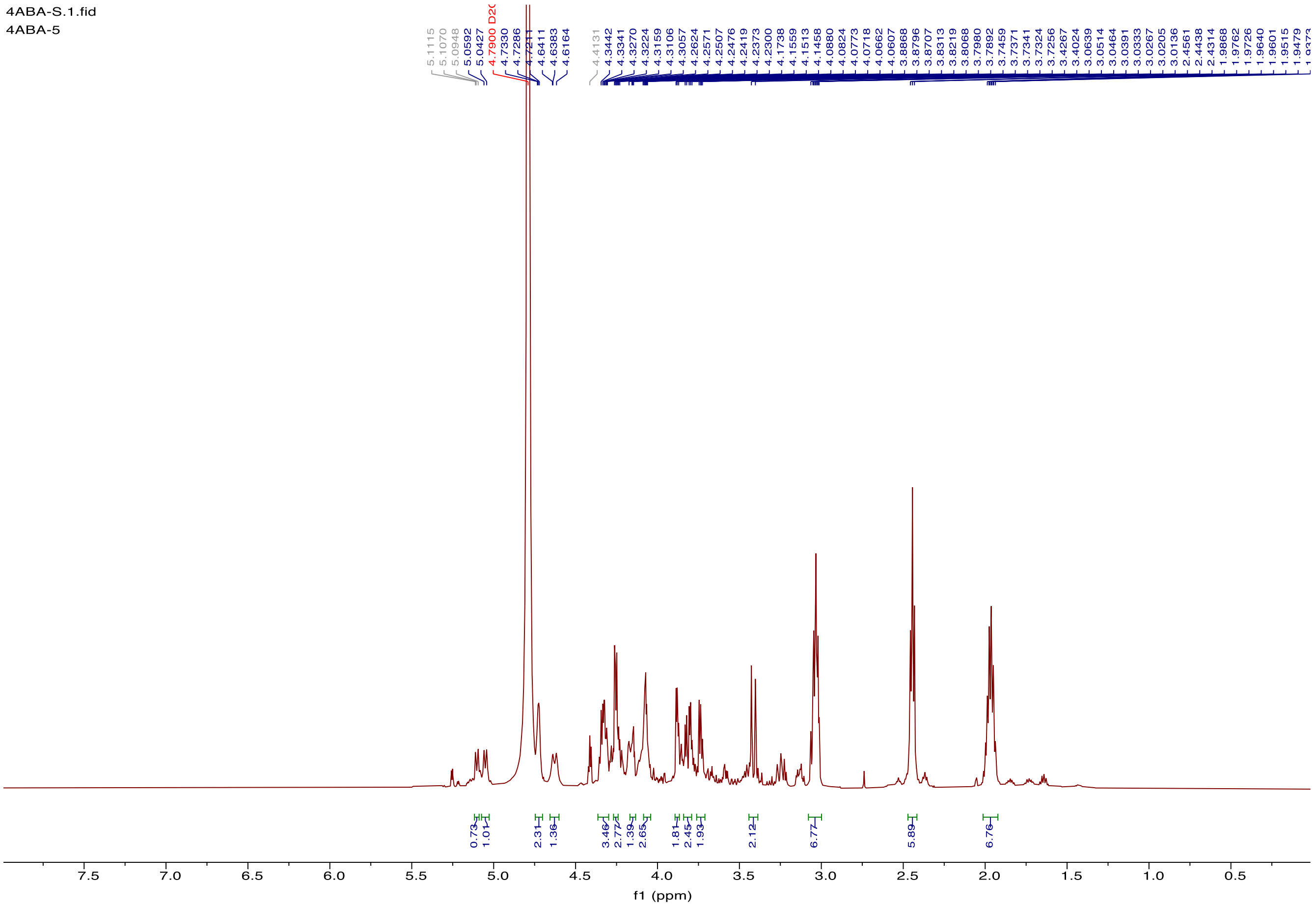

Supplementary Figure 26. ^{1}H NMR of 4-aminobutylthricin in D_2O (600 MHz)

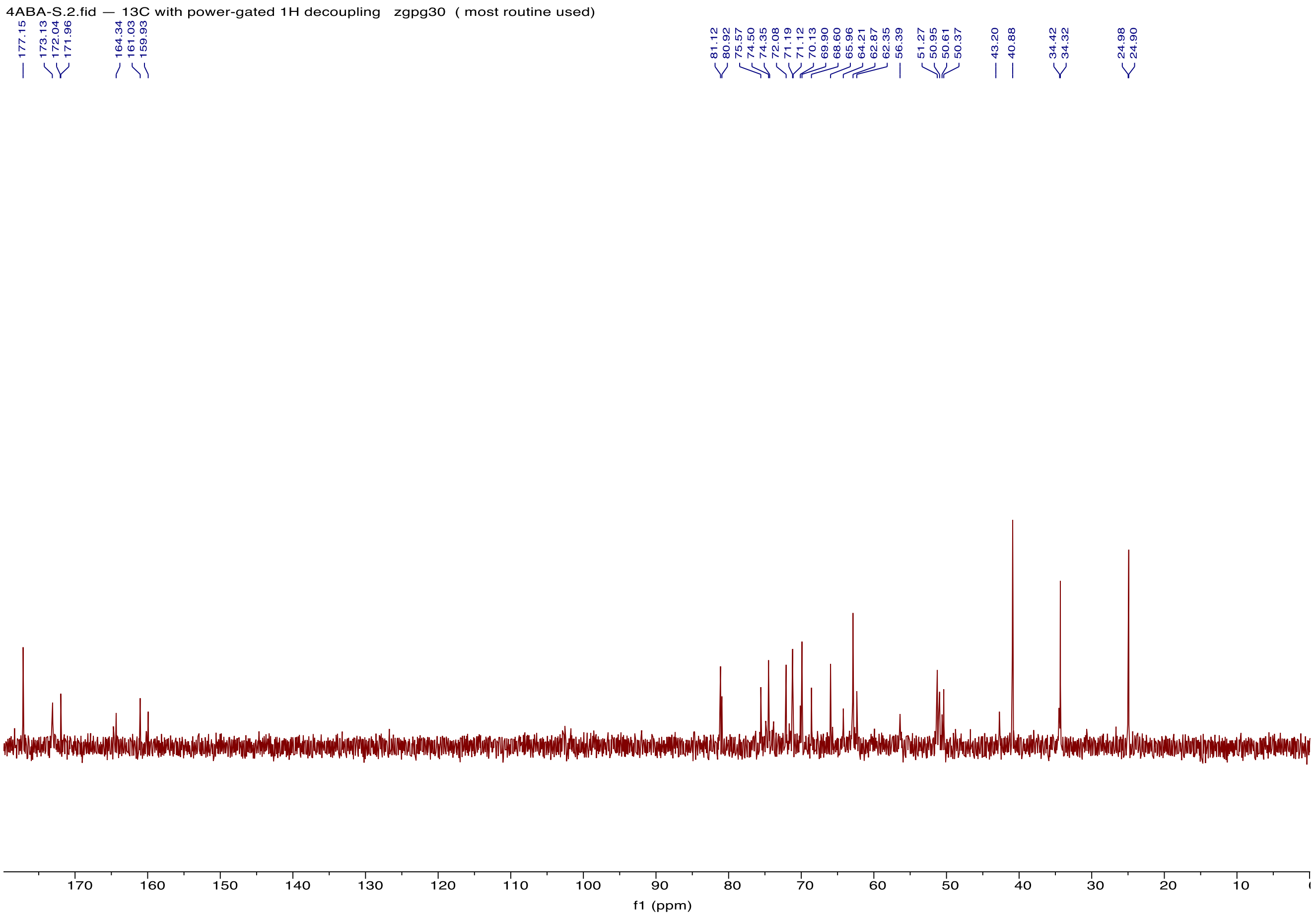

Supplementary Figure 27. ^{13}C **NMR of 4-aminobutylthricin in** D_2O **(150 MHz)**

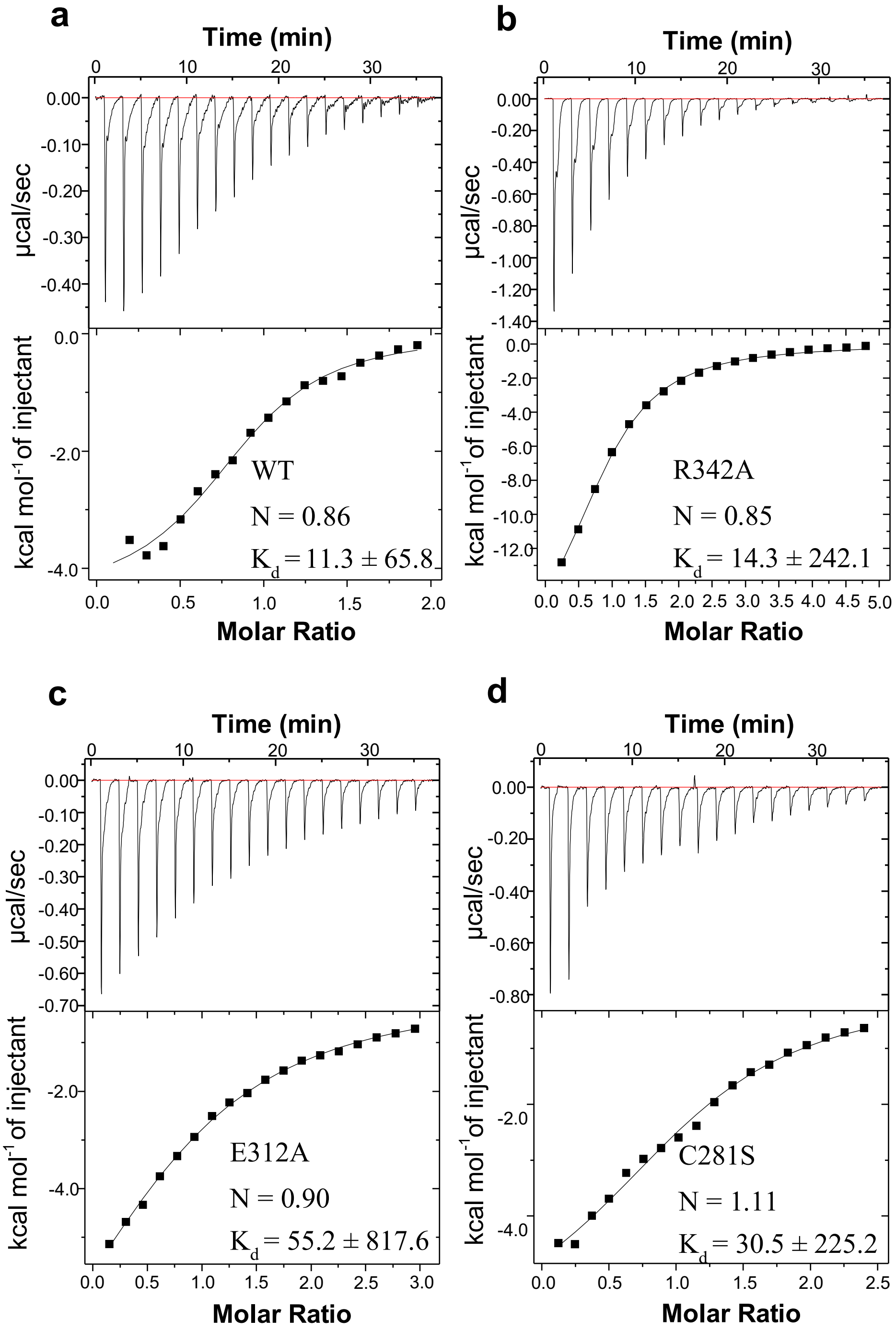

Supplementary Figure 28. ITC binding affinity analysis for Orf1 and mutants versus ST-F. ITC thermographs are shown for ST-F versus (a) wild-type Orf1, (b) R342A, (c) E312A, and (d) C281S.

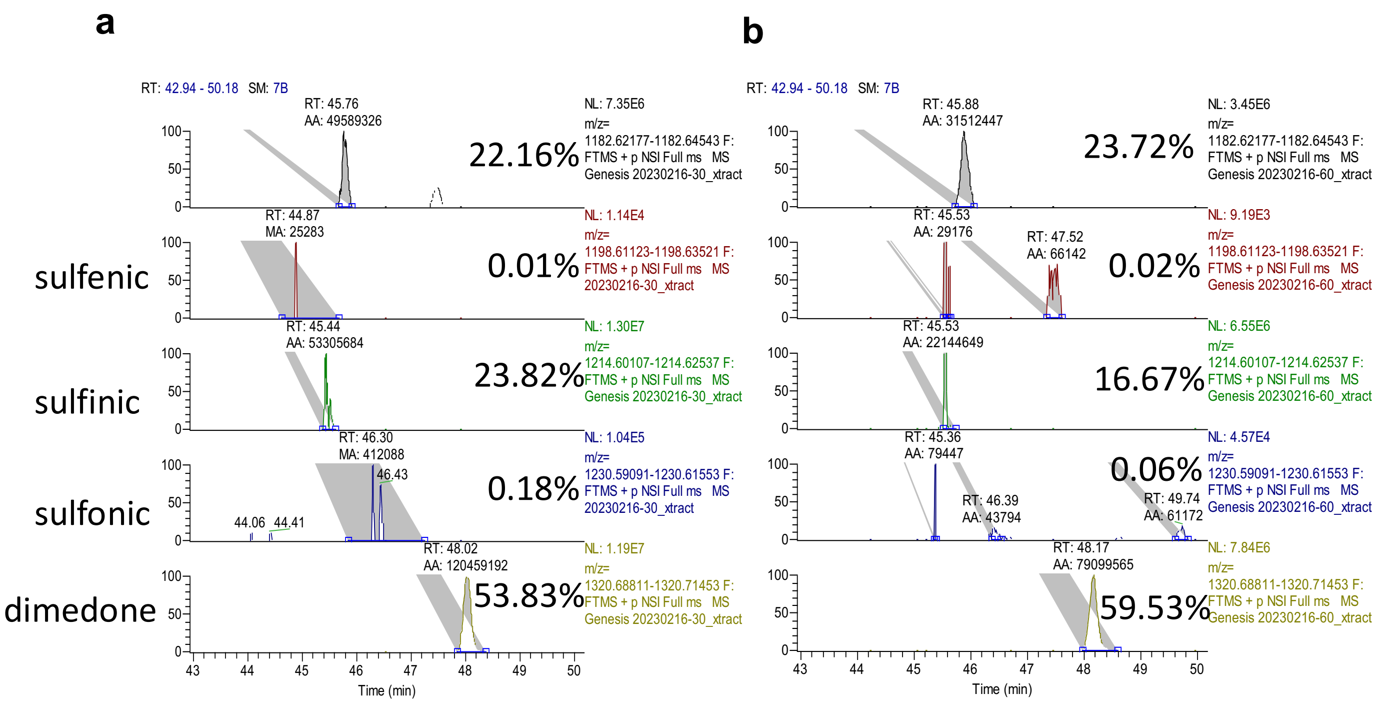

Supplementary Figure 29. Mass spectrometric analyses of the oxidation state of Cys281 in the AFAC^{281}GLHLVPR peptide fragment. Orf1 (50 nM) was incubated in a solution with 250 nM glycine for 30 seconds (a) and 60 seconds (b), respectively. To stop the reactions, 100 mM *N*-acetylglycine (a known inhibitor)[1] was added, followed by addition of 40 mM dimedone to derivatize the sulfenic peptide if any. The samples were then subjected to overnight trypsin digestion and mass spectrometry analysis. The ratio of the unchanged, sulfenic, sulfinic, sulfonic and dimedone peptides was estimated based on their individual peak areas, confirming that the conversion rate for SH-Cys281 to SOH-Cys281 is fast and high. The mass spectrometry file was deposited in ProteomXchange with PXD041104.

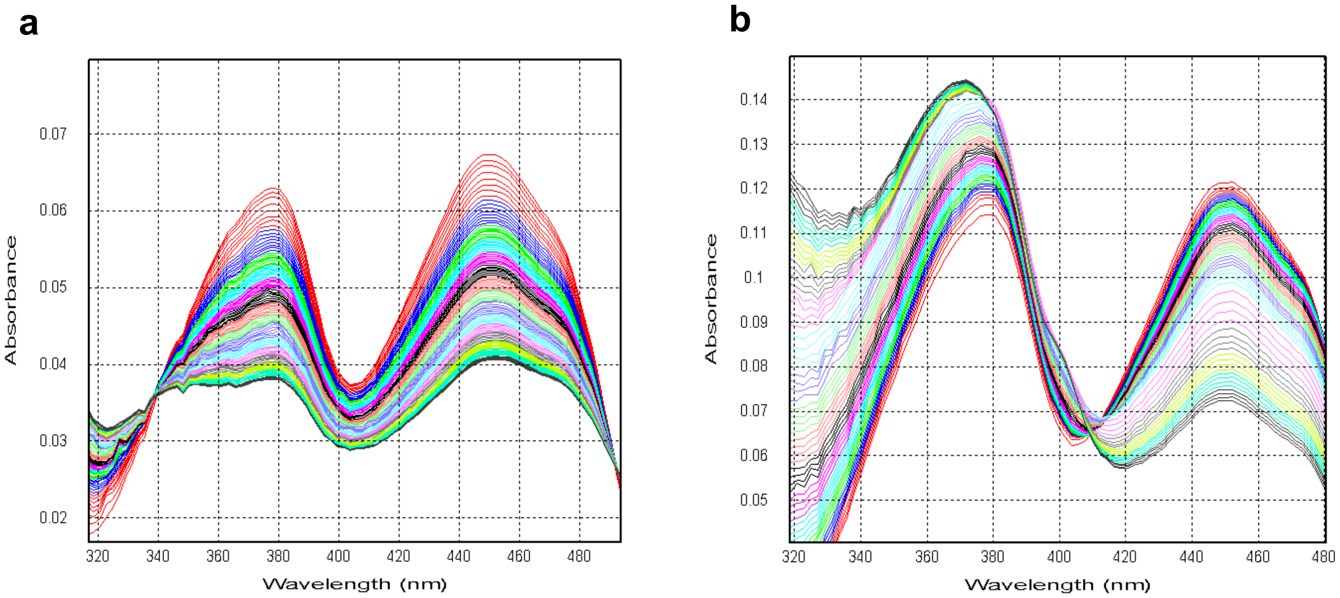

Supplementary Figure 30. UV-Vis spectra of Orf1 added with glycine. The spectra were recorded using a stopped-flow-PDA instrument when Orf1 was mixed with glycine in either (a) an anaerobic or (b) an aerobic buffer solution. The oxidoreduction status of FAD_{ox} is changed over time as shown by PDA spectra in a given color within different time frames. One can observe that FAD_{ox} (with two characteristic peaks at 370 and 450 nm) is gradually reduced over time in both conditions (a) and (b), while in the latter, a bulge emerges on a peak shoulder at 395-405 nm, indicating the formation of a FAD-peroxide species in favor of Orf1 a FPMO.

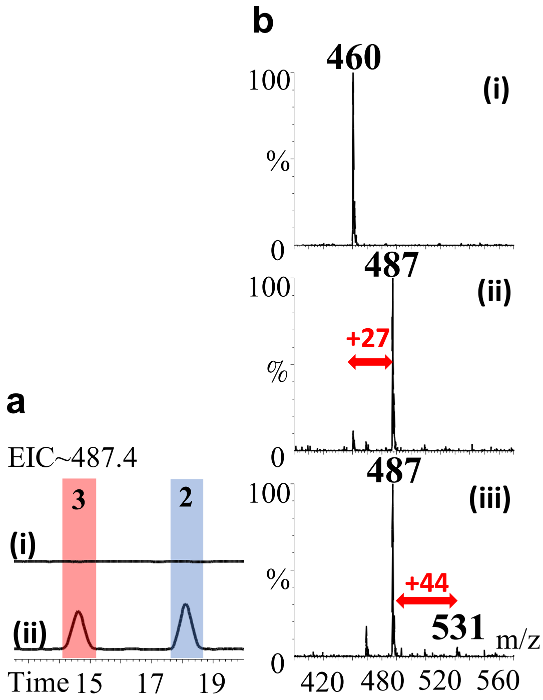

Supplementary Figure 31. Orf1-mediated reactions in the presence of glycinothricin and glycylthricin. (a) The EIC traces of *N*-formimidoyl-glycinothricin (Fig. 1a, compound **2**) (*m/z* 487.4 $[M+H]^+$) for reactions catalyzed by (i) denatured Orf1, (ii) Orf1 with glycine. (b) Mass spectra of (i) **1,** (ii) **2** or (iii) **3**. (c) The EIC traces of **5** (*m/z* 459.2 $[M+H]^+$) for reactions catalyzed by (i) denatured Orf1, (ii) Orf1 in 100 mM MES pH 6.5, (iii) Orf1 in 100 mM HEPES pH 7.0 or (vi) Orf1 in 100 mM HEPES pH 7.5. Source data are provided as a Source Data file.

Supplementary Table 1. Assignments of ^{13}C (150 MHz) and ^{1}H (500 MHz) NMR data[a] for glycinothricin.

position	δ_C	δ_H (multiplicity, J = Hz)
1	170.4	
2	63.4	4.11 (d, 15.1)
3	57.4	[b]4.66
4	64.2	[b]4.73
5	60.4	3.50 (d, 14.5)
		3.82 (dd, 14.5, 5.3)
6	165.5	
7	79.8	5.54 (br)
8	43.5	4.06
9	70.9	4.25 (s)
10	73.6	[b]4.71
11	76.8	4.36 (t, 5.9)
12	63.1	3.72 (d, 5.9)
13	160.8	
14	171.0	
15	41.7	2.72 (s)
16	36.0	2.90 (s)
17	34.8	3.04 (s)

[a] NMR spectra were obtained with the Burker AMX-500 in D_2O including 0.1 % TMSP (trimethylsilyl propanoic acid), and the TMSP peak was used as an internal standard (δ_H 0.0 ppm).

[b] This peak was overlapped with water signal (δ_H 4.75 ppm).

Supplementary Table 2. Assignments of ^{13}C (150 MHz) and ^{1}H (500 MHz) NMR data[a] for iminoacetyl-ST-F.

position	δ_C	δ_H (multiplicity, J = Hz)
1	172.7	
2	63.7	3.95 (d, 10.1)
3	57.2	4.51 (m)
4	63.6	[b]4.60
5	52.1	3.28 (d, 5.6)
		3.69 (dd, 5.6, 14.9)
6	165.9	
7	81.7	4.99 (d, 9.7)
8	51.7	4.15 (dd, 2.9, 9.8)
9	69.3	4.04 (m)
10	72.8	[b]4.68
11	76.3	4.20 (m)
12	63.1	3.62 (d, 6.0)
13	165.5	
14	174.9	
15	39.1	2.57 (dd, 8.2, 16.5)
		2,65 (dd, 4.3, 16.6)
16	51.1	3.56 (m)
17	32.1	1.67 (m)
18	25.7	1.67 (m)
19	44.5	3.30 (m)
20	160.7	
21	162.4	

[a] NMR spectra were obtained with the Burker AMX-500 in D_2O including 0.1% TMSP (trimethylsilyl propanoic acid), and the TMSP peak was used as an internal standard (δ_H 0.0 ppm).

[b] This peak was overlapped with water signal (δ_H 4.75 ppm).

Supplementary Table 3. Biochemical activities of Orf1 and mutants.

Protein	Production of **8** (%)[1]	Production of **6**/**5** (%)[2]	Relative activity of glycine oxidation (%)[3]	K_d with **7** (uM)[4]
WT	100	100/100	100 ± 7.2	11.3 ± 65.8
R342A	N.D.	N.D.	N.D.	14.3 ± 242.1
C281S	N.D.	N.D.	79.1 ± 3.4	30.5 ± 225.2
E426Q	N.D.	N.D.	78.5 ± 6.8	
E312A	N.D.	123/14	81.7 ± 5.5	55.2 ± 817.6
F316A	358	144/0		
WT_DTT	10		11.4 ± 3.5	

[1]The quantity of **8** was estimated according to the peak high of EIC at 574.2. 2.5 μM enzyme was added in a reaction solution containing 50 μM **7**, 0.1 mM glycine, 100 mM sodium phosphate pH 8 and incubated at 37°C for 1 hr.

[2]The quantities of **5** and **6** were estimated according to the peak high of EIC at 459.2. 2.5 μM enzyme was added in a reaction solution containing 60 μM **4**, 0.12 mM glycine, 100 mM sodium phosphate pH 8 and incubated at 37°C for 1 hr.

[3]The same method was applied to enzyme kinetic assay of glycine oxidation. The relative activity of wild-type and mutants were estimated in a reaction condition containing 20 μM enzyme, 2 mM glycine, 100 mM sodium phosphate pH 8 and performed at 37°C within 1 min.

[4]The binding affinities of Orf1 or mutants thereof against ST-F **7** were estimated using isothermal titration calorimetry analysis (ITC) (Supplementary Fig. 28).

Supplementary Table 4. Primers used for gene cloning and mutagenesis.

	Primer sequences (5′--3′)	experiments
Forward	(orf1_loxP_F) **gatgtgcgagtgactctcctgggaaagcccgcgcgccagtacggcgcg**ttcgagcgactcgagt	*orf1* inactivation homologous region of the orf1 gene (bold)
Reverse	(orf1_loxP_R) **gtaggtgtcgctcagccggtccgcgagctcggcgaacgggccctggagga**ggtaccgagcgaacgcgtt	
Forward	ctagctagcatgaaaaggcattatgaagcagtggtgattgga	*thiO* gene cloning
Reverse	ccgctcgagtatctgaaccgcctccttgcgatcaatt	
Forward	gattacatatgaccacgacccatgacacg	*sttE* gene cloning
Reverse	gattaaagctttcaggggcagggcatgctcatg	
Forward	ccgcgccttcgccgccggactgcacctg	Orf1-C281A
Reverse	caggtgcagtccggcggcgaaggcgcgg	
Forward	gcgccttcgccagcggactgcac	Orf1-C281S
Reverse	gtgcagtccgctggcgaaggcgc	
Forward	gcggccagcatcgcggagacggtcttc	Orf1-E312A
Reverse	gaagaccgtctccgcgatgctggccgc	
Forward	ggcgcagttgaagagggcgaccgtctcctcgatg	Orf1-F316A
Reverse	catcgaggagacggtcgccctcttcaactgcgcc	
Forward	gtgcaggtgggcagtgccccggcc	Orf1-R342A
Reverse	ggccggggcactgcccacctgcac	
Forward	gcgacgggctaccagttcccctggc	Orf1-E426Q
Reverse	gccaggggaactggtagcccgtcgc	
Forward	cctgcggactggccctggtgcccc	Orf1-H284A
Reverse	ggggcaccagggccagtccgcagg	
Forward	gcggttcggtcttcatcggcgccac	Orf1-Y294F

Reverse	gtggcgccgatgaagaccgaaccgc	
Forward	cgtgggccattccgctgatgacgacgcgaatttcg	Orf1-L124D
Reverse	cgaaattcgcgtcgtcatcagcggaatggcccacg	
Forward	cgtgggccattccgctgaggacgacgcga	Orf1-L124E
Reverse	tcgcgtcgtcctcagcggaatggcccacg	
Forward	cctccaaccgcgtcttcgcctgcgg	Orf1-A278V
Reverse	ccgcaggcgaagacgcggttggagg	
Forward	caaccgcgccttcagctgcggactgcac	Orf1-A280S
Reverse	gtgcagtccgcagctgaaggcgcggttg	
Forward	cgacgtggtccggtataccatggcgacgg	Orf1-H419Y
Reverse	ccgtcgccatggtataccggaccacgtcg	
P100	atcttgctgaaaaactcgagccatccgg	*orfV*-deficient mutant
P101	atctttctagaagatctcctacaatattctcagctg	
P223	attattattattaagcttgaataggaacttcggaataggaacttatga	
P224	attattattattaagcttaagtataggaacttcgaagttcccg	
P01	attattgctagcgaacccgaccgacctttggggactgacatg	
P02	attattctcgagtgcgaacagctcgcgcaactgcacccgta	
P03	attctcgagtggacgcgtccgcatccggtcgaggtgat	
P04	atttctagagtccacggaagaggtggggtcctccagga	
P05	cacctcgttcaacggccctgacgaaccg	
P06	accgaggcgtcgaagaggcggttggtca	

Supplementary Table 5. ^{1}H (600 MHz) and ^{13}C (150 MHz) NMR data of glycylthricin in D_2O

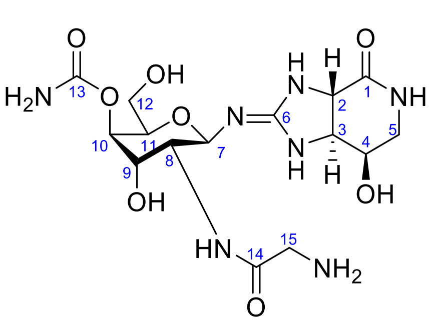

10-carbamoyl glycylthricin

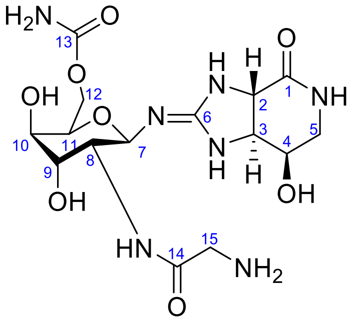

12-carbamoyl glycylthicin

	10-carbamoyl **4**		12-carbamoyl **4**	
No.	δ_H (mult, J in Hz)	δ_C (type)	δ_H (mult, J in Hz)	δ_C (type)
1		172.1 (C)		172.1 (C)
2	4.62 (d, 14.2)	56.4 (CH)	4.62 (d, 14.2)	56.4 (CH)
3	4.07 (dd, 3.4, 14.2)	62.9 (CH)	4.07 (dd, 3.4, 14.6)	62.9 (CH)
4	4.72 (overlapping)	62.9 (CH)	4.72 (overlapping)	62.9 (CH)
5	3.41 (d, 14.6)	51.2 (CH_2)	3.41 (d, 14.6)	51.3 (CH_2)
	3.81 (dd, 5.6, 14.6)		3.81 (dd, 5.6, 14.6)	
6		164.9 (C)		164.5 (C)
7	5.10 (d, 9.8)	80.9 (CH)	5.06 (d, 9.8)	81.0 (CH)
8	4.25 (dd, 3.6, 9.8)	50.6 (CH)	4.25 (dd, 3.6, 9.8)	50.6 (CH)
9	4.18 (t, 3.6)	68.5 (CH)	4.10 (t, 3.4)	69.9 (CH)
10	4.72 (overlapping)	72.1 (CH)	3.88 (d, 3.5)	71.1 (CH)
11	4.34 (overlapping)	75.6 (CH)	4.34 (overlapping)	74.5 (CH)
12	3.73 (br d, 6.5)	62.3 (CH_2)	4.25 (overlapping)	65.9 (CH_2)
	3.73 (br d, 6.5)		4.25 (overlapping)	
13		159.9 (C)		161.0 (C)
14		173.2 (C)		169.8 (C)
15	3.84 (d, 6.9)	42.7 (CH_2)	3.84 (d, 6.9)	42.7 (CH_2)
	3.84 (d, 6.9)		3.84 (d, 6.9)	

Supplementary Table 6. ^{1}H (600 MHz) and ^{13}C (150 MHz) NMR data of ST-F in D_2O

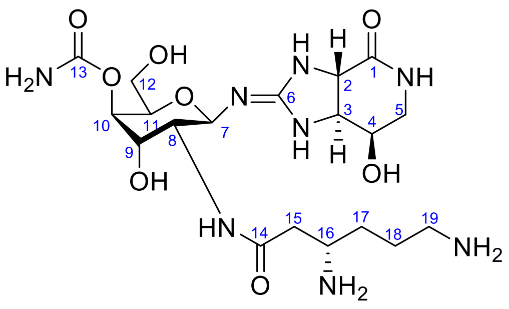

ST-F

No.	δ_H (mult, J in Hz)	δ_C (type)
1		172.0 (C)
2	4.63 (d, 14.6)	56.4 (CH)
3	4.08 (d, 14.6)	62.9 (CH)
4	4.72 (overlapping)	62.9 (CH)
5	3.40 (d, 14.6)	51.3 (CH_2)
	3.81 (dd, 5.6, 14.6)	
6		164.8 (C)
7	5.11 (d, 9.8)	80.8 (CH)
8	4.25 (dd, 4.1, 9.8)	50.9 (CH)
9	4.17 (t, 4.1)	68.5 (CH)
10	4.72 (overlapping)	72.1 (CH)
11	4.33 (t, 6.3)	75.5 (CH)
12	3.73 (d, 6.1)	62.3 (CH_2)
	3.73 (d, 6.1)	
13		160.0 (C)
14		174.0 (C)
15	2.69 (dd, 8.1, 16.8)	38.3 (CH_2)
	2.80 (dd, 4.2, 16.8)	
16	3.68 (m)	50.2 (CH)
17	1.79 (m)	31.0 (CH_2)
	1.79 (m)	
18	1.79 (m)	24.9 (CH_2)
	1.79 (m)	
19	3.04 (m)	40.9 (CH_2)
	3.04 (m)	

Supplementary Table 7. ^{1}H (600 MHz) and ^{13}C (150 MHz) NMR data of 3-aminopropionylthricin in D_2O

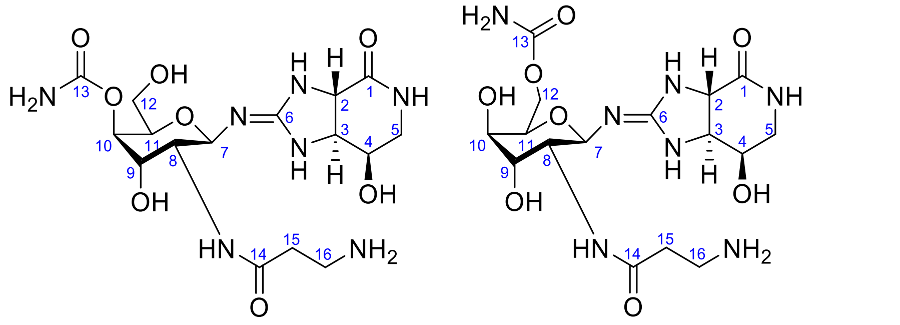

10-carbamoyl 3-aminopropionylthricin 12-carbamoyl 3-aminopropionylthricin

	10-carbamoyl **9**		12-carbamoyl **9**	
No.	δ_H (mult, *J* in Hz)	δ_C (type)	δ_H (mult, *J* in Hz)	δ_C (type)
1		172.1 (C)		172.0 (C)
2	4.63 (d, 14.3)	56.4 (CH)	4.63 (d, 14.3)	56.4 (CH)
3	4.07 (dd, 3.6, 14.3)	62.9 (CH)	4.07 (dd, 3.6, 14.3)	62.9 (CH)
4	4.73 (overlapping)	62.9 (CH)	4.73 (overlapping)	62.9 (CH)
5	3.41 (d, 14.7)	51.0 (CH_2)	3.41 (d, 14.7)	51.3 (CH_2)
	3.81 (dd, 5.7, 14.7)		3.81 (dd, 5.7, 14.7)	
6		164.8 (C)		164.5 (C)
7	5.10 (d, 9.9)	80.9 (CH)	5.06 (d, 9.9)	81.0 (CH)
8	4.26 (dd, 3.4, 9.9)	50.4 (CH)	4.26 (dd, 3.6, 9.9)	50.4 (CH)
9	4.17 (t, 3.4)	68.5 (CH)	4.08 (t, 3.6)	69.9 (CH)
10	4.73 (overlapping)	72.1 (CH)	3.88 (br d, 3.7)	71.1 (CH)
11	4.33 (overlapping)	75.6 (CH)	4.33 (overlapping)	74.4 (CH)
12	3.74 (dd, 3.7, 6.4)	62.3 (CH_2)	4.26 (overlapping)	65.9 (CH_2)
	3.74 (dd, 3.7, 6.4)		4.26 (overlapping)	
13		159.9 (C)		161.0 (C)
14		173.1 (C)		174.5 (C)
15	2.75 (td, 3.6, 6.7)	34.0 (CH_2)	2.75 (td, 3.6, 6.7)	33.8 (CH_2)
	2.75 (td, 3.6, 6.7)		2.75 (td, 3.6, 6.7)	
16	3.29 (m)	37.8 (CH_2)	3.29 (m)	37.6 (CH_2)

Supplementary Table 8. ^{1}H (600 MHz) and ^{13}C (150 MHz) NMR data of 4-aminobutylthricin in D_2O

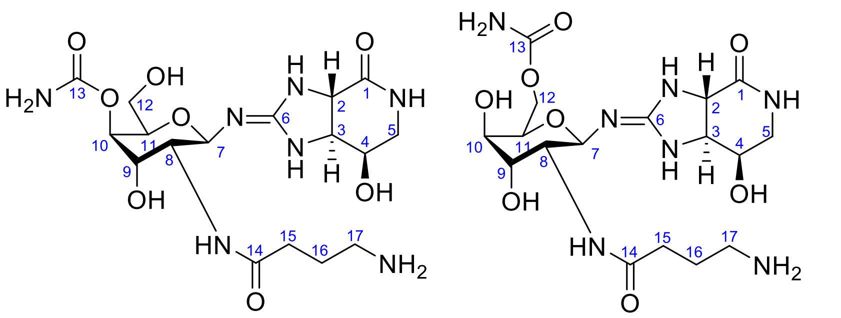

10-carbamoyl 4-aminobutylthricin 12-carbamoyl 4-aminobutylthricin

	10-carbamoyl **10**		12-carbamoyl **10**	
No.	δ_H (mult, J in Hz)	δ_C (type)	δ_H (mult, J in Hz)	δ_C (type)
1		172.0 (C)		172.0 (C)
2	4.63 (d, 14.8)	56.4 (CH)	4.63 (d, 14.8)	56.4 (CH)
3	4.07 (dd, 3.4, 14.8)	62.9 (CH)	4.07 (overlapping)	62.9 (CH)
4	4.73 (overlapping)	62.9 (CH)	4.73 (overlapping)	62.9 (CH)
5	3.41 (d, 14.6)	51.0 (CH_2)	3.41 (d, 14.6)	51.3 (CH_2)
	3.81 (dd, 5.5, 14.6)		3.81 (dd, 5.5, 14.6)	
6		164.7 (C)		164.3 (C)
7	5.11 (d, 9.9)	80.9 (CH)	5.05 (d, 9.9)	81.1 (CH)
8	4.25 (dd, 3.6, 9.8)	50.4 (CH)	4.25 (dd, 3.5, 9.8)	50.4 (CH)
9	4.15 (t, 3.6)	68.6 (CH)	4.07 (t, 3.5)	69.9 (CH)
10	4.73 (overlapping)	72.1 (CH)	3.88 (d, 3.5)	71.2 (CH)
11	4.32(overlapping)	75.6 (CH)	4.32 (overlapping)	74.5 (CH)
12	3.74 (dd, 5.2, 7.0)	62.4 (CH_2)	4.25 (overlapping)	66.0 (CH_2)
	3.74 (dd, 5.2, 7.0)		4.25 (overlapping)	
13		159.9 (C)		161.0 (C)
14		173.1 (C)		177.2 (C)
15	2.44 (t, 7.4)	34.4 (CH_2)	2.44 (t, 7.4)	34.3 (CH_2)
	2.44 (t, 7.4)		2.44 (t, 7.4)	
16	1.96 (m)	25.0 (CH_2)	1.96 (m)	24.9 (CH_2)
	1.96 (m)		1.96 (m)	
17	3.03 (ddd, 3.6, 7.6, 11.8)	40.9 (CH_2)	3.03 (ddd, 3.6, 7.6, 11.8)	40.9 (CH_2)
	3.03 (ddd, 3.6, 7.6, 11.8)		3.03 (ddd, 3.6, 7.6, 11.8)	

Supplementary Methods

Heterologous expression of BD-12 and its *orf1*-deficient BGC

A DNA fragment containing the a *loxP-aphII-loxP* region of pKU479[(ref2)] was amplified by PCR using two PCR primers, orf1-loxP-F (forward, 64 nt) and orf1-loxP-R (reverse, 69 nt), which share the homologous regions (48 nt for orf1-loxP-F and 50 nt for orf1-loxP-R) of the *orf1* gene (Supplementary Table 4). The amplified DNA fragment was introduced into *E. coli* BW25113 carrying pKD46 and pRED-BD-12cluster[2] to perform an *in vivo* gene replacement reaction mediated by λ-Red recombinase. The resulting plasmid, pRED-BD-12clusterΔorf1::loxP-aphII-loxP, was obtained, and the marker gene (aphII) was removed by an *in vitro* loxP homologous recombination using Cre recombinase (New England Biolabs, USA). The resulting plasmid, pRED-BD-12clusterΔorf1, in which the *orf1* gene was inactivated by in-frame deletion, was digested with SpeI and NheI. A 34-kbp DNA fragment was obtained and cloned into a *Streptomyces* integrating vector, pKU493A_aac(3)IV[3], to generate pKU493A-BD-12Δorf1. The constructed integration vector, pKU493A-BD-12Δorf1, was introduced into a heterologous host strain, *S. avermitilis* SUKA17, by standard procedures[4]. The *S. avermitilis* SUKA17 transformant harboring pKU493-BD12Δorf1 was cultured in AVM medium containing 6% (w/v) glucose, 0.2% (w/v) yeast extract (Difco Laboratories, Franklin Lakes, NJ, USA), 0.2% (w/v) $(NH_4)SO_4$, 0.5% (w/v) $CaCO_3$, 0.2% (w/v) NaCl, 0.05% (w/v) K_2HPO_4, 0.01% (w/v) $MgSO_4·7H_2O$, 0.005% (w/v) $FeSO_4·7H_2O$, 0.005% (w/v) $ZnSO_4·7H_2O$, and 0.005% (w/v) $MnSO_4·4H_2O$ (pH 7.0) for 6 days at 28°C.

Purification of glycinothricin

The culture broth (1,400 ml) of the *S. avermitilis* SUKA17 transformant harboring pKU493A-BD-12Δorf1 was centrifuged, and the supernatant obtained was mixed with 1,400 ml of chloroform. After vigorous shaking, the aqueous layer after centrifugation was adjusted to pH 5.0 using acetic acid. This sample was mixed with 200 ml acetonitrile and centrifuged. The supernatant was loaded onto a Dowex 50W×2 column (100 to 200 mesh; H^+ form; 10 × 14 cm; Dow Chemical, Midland, MI, USA), and the column was washed with 200 ml of 40% (v/v) acetonitrile in water. The sample was eluted in a stepwise fashion with 420 ml of 0.1, 0.2, 0.4, 0.6, 0.8, 1 and 1.6 M KCl in 40% (v/v) acetonitrile. The glycinothricin (Fig. 1a, compound **1**) fractions eluted with 0.4 and 0.6 M KCl were combined. After removing the organic solvent *in vacuo*, the aqueous layer was lyophilized to give a white powder. This sample was dissolved in a small volume of methanol, the insoluble materials were removed by centrifugation, and the methanol-soluble fraction was evaporated *in vacuo*. The dried sample was dissolved in a small volume of water, and then was applied to an activated-carbon column (2.0 × 10 cm) that had been equilibrated with water. The column was washed with water, and the fraction of **1** was eluted with 50% (v/v) acetone in water adjusted to pH 2.0 by HCl. After removing the organic solvent, the aqueous layer was lyophilized to give a white powder. This sample was dissolved in a small volume of water and fractionated by preparative HPLC using a reversed-phase column (Sunniest RP-AQUA; 5 μm; 10 × 250 mm; ChromaNik Technologies) at 40°C at a flow rate of 4.72 ml min^{-1} and with a mobile phase composed of 14% (v/v) acetonitrile and 0.1% (v/v) *n*-heptafluorobutyric acid (HFBA). Fractions were collected and monitored with a UV detector at 210 nm. The fraction containing **1** was lyophilized, dissolved in a small volume of 50% (v/v) acetonitrile in water, and fractionated by preparative HPLC using a hydrophilic- interaction liquid chromatography (HILIC) column (ZIC-HILIC; 5 μm; 10 × 250 mm; Merck, Kenilworth, NJ, USA) at 55°C at a flow rate of 8 ml min^{-1} and with a mobile

phase composed of 60% (v/v) acetonitrile, 5 mM $HCOONH_4$, and 0.1% (v/v) formic acid. Fractions were collected and monitored with a UV detector at 210 nm. The fraction containing **1** was lyophilized to give a white powder. This sample was dissolved in a small volume of water and fractionated by preparative HPLC using a reversed-phase column under the conditions described above. The fraction containing **1** was lyophilized to obtain the highly purified **1** (approximately 6.4 mg), whose chemical structure was then determined by nuclear magnetic resonance (NMR) analysis.

Purification of *N*-iminoacetyl-ST-F

A reaction mixture (100 ml) consisting of 50 mM CAPS (pH 9.0), 0.3 mM ST-F, 0.3 mM glycine and 140 μg ml^{-1} Orf1 was incubated at 40°C for 5 h. The reaction mixture was mixed with 100 ml chloroform. After vigorous shaking, the aqueous layer from centrifugation was adjusted to pH 5 using acetic acid. This sample was applied to an activated-carbon column (1.0 × 10 cm) that had been equilibrated with water. The column was washed with water, and the *N*-iminoacetyl-ST-F (Fig. 1a, compound **8**) fraction was eluted with 50% (v/v) acetone in water adjusted to pH 2 by HCl. After the removal of the organic solvent *in vacuo*, the aqueous layer was lyophilized to give a white powder. This sample was dissolved in a small volume of water and fractionated by preparative HPLC using a reversed-phase column (Sunniest RP-AQUA; 5 μm; 10 × 250 mm; ChromaNik Technologies) at 40°C at a flow rate of 4.72 ml min^{-1} and with a gradient of acetonitrile-water run over 19 min (8% (v/v) acetonitrile for 2 min, 8-15% (v/v) acetonitrile for 17 min). Both acetonitrile and water contained 0.1% (v/v) HFBA. Fractions were collected and monitored with a UV detector at 210 nm. The fraction containing **8** was lyophilized to obtain the highly purified compound (approximately 4.4 mg), whose chemical structure was then determined by NMR analysis.

Constructing an *orfV*-deficient mutant

A suicide plasmid WZC02 was constructed for knocking out the *orfV* gene in the genome of *Streptomyces lavendulae* BCRC 12163[(ref5)]. In brief, the DNA backbone amplified from pJET1.2/blunt cloning vector (Thermo Fisher) with 5'-phosphorylated primers, P100 and P101 were self-ligated using T4 DNA ligase (Thermo Fisher). The resultant plasmid was digested and then ligated with the DNA fragment, which were amplified from plasmid pGUSRolRPA3 using P223 and P224, within HindIII restriction site[12]. The resulting plasmid, WZC02, was applied to knock out the specific genes of *S. lavendulae* BCRC 12163 using an allelic-exchange strategy. DNA fragments flanking both ends of *orfV* gene were amplified by PCR with primer sets P01, P02 and P03, P04, respectively. The amplicons were individually cloned into the suicide vector WZC02. The plasmid resulted, WZC02-*orfV*, was transformed into *E. coli* ET12567 (pUZ8002) and then mobilized to the streptothricin producing strain *S. lavendulae* BCRC 12163[(ref6)]. Individual apramycin-resistant transconjugant was cultured on selective plates and confirmed using primer set P05 and P06. After a second homologous recombination event, the apramycin-sensitive colonies were isolated and the *orfV*-deficient mutants were verified using polymerase chain reaction (PCR) with primers P05 and P06. The *orfV* gene is a nonribosomal peptide-synthetase (NRPS) containing thiolation and condensation domains for the condensation of L-β-lysine and streptothrisamine[7]. The *orfV*-deficient mutant can give rise to accumulation of streptothrisamine in culture.

Preparation of streptothrisamine

Streptothrisamine was produced from the *orfV*-deficient strain of *Streptomyces lavendulae* BCRC 12163 and purified by using the same protocol reported previously[5,7]. The *orfV*-deficient mutant was cultured in the medium containing 2% maltose, 0.5% tryptone, 0.5% meat extract, 0.3% yeast extract, 0.3% NaCl, 0.1% $MgSO_4$, 0.005% $FeSO_4 \cdot 7H_2O$, 0.005% $ZnSO_4 \cdot 7H_2O$, and 0.005% $MnSO_4 \cdot 4H_2O$ (pH 7.4) for 7 days at 28°C. The supernatant from the centrifuged culture broth was mixed with chloroform and vigorously shaken. The aqueous layer was combined with Diaion HP-20 and the resulting suspension was filtered. The filtrate was loaded onto a Dowex 50W-X2 column (NH_4^+ form) and washed with water. The sample was eluted with 1 M ammonium bicarbonate and desalted using an activated-carbon column. After lyophilized the elution from the activated-carbon column, the sample was further purified by using a TSKgel® Amide-80 column (5 μm, 4.6 mm x 250 mm) at 30°C at a flow rate of 1 ml min^{-1} and with a gradient of acetonitrile-water run over 30 min (65% (v/v) acetonitrile for 5 min, 65-20% (v/v) acetonitrile for 25 min). Both acetonitrile and water contained 0.1% (v/v) formic acid. Fractions were collected and monitored with a UV detector at 210 nm.

Preparations of compounds 4, 7, 9 and 10

The amine of glycine, 3-aminopropionate and 4-aminobutyrate were protected by benzyl-carbamate (cbz) and activated by EDC and NHS (*N*-ethyl-*N*'-(3-(dimethylamino)propyl)carbodiimide/*N*-hydroxylsuccinimide) at a 1:2:2 ratio. The streptothrisamine was mixed with cbz-glycine-NHS, cbz-3-aminopropionic-NHS or cbz-4-aminobutyric-NHS in DMSO at a 1:1.2 ratio overnight at room temperature. The products were purified by using a C18 column (4.6 x 250 mm, 5 μm, Prodigy, Phenomenex) at 30°C at a flow rate of 1 ml min^{-1} and with a gradient of acetonitrile-water run over 30 min (2% (v/v) acetonitrile for 5 min, 2-98% (v/v) acetonitrile for 25 min). The cbz-glycylthricin, cbz-3-aminopropionylthricin and cbz-4-aminobutylthricin were deprotected by Pd/C catalysts from $Pd(OAc)_2$ and charcoal in methanol. The mixture of **7** and ST-D was purchased from GoldBio. Compound **7** was further purified by using a TSKgel® Amide-80 column (5 μm, 4.6 mm x 250 mm) at 30°C at a flow rate of 1 ml min^{-1} and with a gradient of acetonitrile-water run over 30 min (65% (v/v) acetonitrile for 5 min, 65-20% (v/v) acetonitrile for 25 min). Both acetonitrile and water contained 0.1% (v/v) formic acid. Compounds **4, 7**, **9** and **10** were identified by NMR analysis or complex crystal structures with Orf1.

Supplementary References

1 Settembre, E. C. *et al*. Structural and mechanistic studie on ThiO, a glycine oxidase essential for thiamin biosynthesis in Bacillus subtilis. *Biochemistry* **42**, 2971-2981, doi:10.1021/bio026916v (2003).

2 Komatsu, M., Uchiyama, T., Omura, S., Cane, D. E. & Ikeda, H. Genome-minimized *Streptomyces* host for the heterologous expression of secondary metabolism. *Proc. Natl. Acad. Sci. USA* **107**, 2646-2651, doi:10.1073/pnas.0914833107 (2010).

3 Maruyama, C. *et al.* tRNA-dependent aminoacylation of an amino sugar intermediate in the biosynthesis of a streptothricin-related antibiotic. *Appl. Environ. Microbiol.* **82**, 3640-3648, doi:10.1128/aem.00725-16 (2016).

4 Komatsu, M. *et al.* Engineered *Streptomyces avermitilis* host for heterologous expression of biosynthetic gene cluster for secondary metabolites. *ACS Synth. Biol.* **2**, 384-396, doi:10.1021/sb3001003 (2013).

5 Chang, C. Y. *et al.* Biosynthesis of streptolidine involved two unexpected intermediates produced by a dihydroxylase and a cyclase through unusual mechanisms. *Angew. Chem. Int. Ed. Engl.* **53**, 1943-1948, doi:10.1002/anie.201307989 (2014).

6 Horbal, L., Fedorenko, V. & Luzhetskyy, A. Novel and tightly regulated resorcinol and cumate-inducible expression systems for *Streptomyces* and other actinobacteria. *Appl. Microbiol. Biotechnol.* **98**, 8641-8655, doi:10.1007/s00253-014-5918-x (2014).

7 Maruyama, C. *et al.* A stand-alone adenylation domain forms amide bonds in streptothricin biosynthesis. *Nat. Chem. Biol.* **8**, 791-797, doi:10.1038/nchembio.1040 (2012).
